# Between the Balkans and the Baltic: Phylogeography of a Common Vole Mitochondrial DNA Lineage Limited to Central Europe

**DOI:** 10.1371/journal.pone.0168621

**Published:** 2016-12-16

**Authors:** Joanna Stojak, Allan D. McDevitt, Jeremy S. Herman, Boris Kryštufek, Jitka Uhlíková, Jenő J. Purger, Leonid A. Lavrenchenko, Jeremy B. Searle, Jan M. Wójcik

**Affiliations:** 1 Mammal Research Institute, Polish Academy of Sciences, Białowieża, Poland; 2 Department of Ecology and Evolutionary Biology, Cornell University, Ithaca, New York, United States of America; 3 Ecosystems and Environment Research Centre, School of Environment and Life Sciences, University of Salford, Salford, United Kingdom; 4 Department of Natural Sciences, National Museums Scotland, Edinburgh, United Kingdom; 5 Vertebrate Department, Slovenian Museum of Natural History, Ljubljana, Slovenia; 6 Nature Conservation Agency of the Czech Republic, Prague, Czech Republic; 7 Department of Ecology, Institute of Biology, University in Pécs, Pécs, Hungary; 8 Department of Mammalian Microevolution, A.N. Severtsov Institute of Ecology and Evolution, Russian Academy of Sciences, Moscow, Russia; Universita degli Studi di Roma La Sapienza, ITALY

## Abstract

The common vole (*Microtus arvalis*) has been a model species of small mammal for studying end-glacial colonization history. In the present study we expanded the sampling from central and eastern Europe, analyzing contemporary genetic structure to identify the role of a potential ‘northern glacial refugium’, i.e. a refugium at a higher latitude than the traditional Mediterranean refugia. Altogether we analyzed 786 cytochrome *b* (cyt*b*) sequences (representing mitochondrial DNA; mtDNA) from the whole of Europe, adding 177 new sequences from central and eastern Europe, and we conducted analyses on eight microsatellite loci for 499 individuals (representing nuclear DNA) from central and eastern Europe, adding data on 311 new specimens. Our new data fill gaps in the vicinity of the Carpathian Mountains, the potential northern refugium, such that there is now dense sampling from the Balkans to the Baltic Sea. Here we present evidence that the Eastern mtDNA lineage of the common vole was present in the vicinity of this Carpathian refugium during the Last Glacial Maximum and the Younger Dryas. The Eastern lineage expanded from this refugium to the Baltic and shows low cyt*b* nucleotide diversity in those most northerly parts of the distribution. Analyses of microsatellites revealed a similar pattern but also showed little differentiation between all of the populations sampled in central and eastern Europe.

## Introduction

Phylogeography is a discipline which uses molecular markers to infer population movements at the end of the Pleistocene and during the Holocene, to identify where glacial refugia were located and to establish genetic characteristics of present-day populations, including geographic barriers obstructing gene flow [1]. Therefore, phylogeography attempts to reconstruct the history of contemporary species distributions and explain the partitioning of genetic variation within them. Europe is one of the most comprehensively studied regions in the context of Pleistocene phylogeography.

During the Pleistocene in Europe there were three substantial glacial periods, separated by warmer interglacials: the Elster glaciation (730–430 kya, thousands of years ago), the Saale glaciation (300–130 kya) and the Weichselian (or Vistulian) glaciation (115–11.5 kya) [2]. Considering the Weichselian, the ice cover in Europe reached its maximum extent around 27.5–19 kya and this time point is known as the Last Glacial Maximum (LGM) [3]. The Scandinavian ice sheet, that dominated northern Europe at the LGM, reached parts of the British Isles, Germany, Poland and Russia, while neighboring regions suffered tundra conditions at this time [4, 5]. More favorable conditions for temperate species were present on the three Mediterranean peninsulas (Iberian, Apennine and Balkan), which played the role of refugial areas, where many species survived cold periods [6–8]. Additionally, there is evidence that some temperate species also survived in more northerly areas, such as the Carpathian Basin, parts of present-day France, the Ural Mountains and the Russian Plains [6, 9–16]. After the LGM there was a warmer phase designated the Bølling-Allerød interstadial (14.7–12.7 kya), when deglaciation occurred [17, 18]. However, the retreat of the glaciers caused many icebergs to discharge into the ocean, disrupting the Atlantic meridional overturning circulation (AMOC) and resulting in further cooling of the climate [19, 20]. Consequently, the Pleistocene ended with a short cold phase, known as the Younger Dryas (YD; 12.9–11.7 kya) [21, 22].

**Fig 1:**
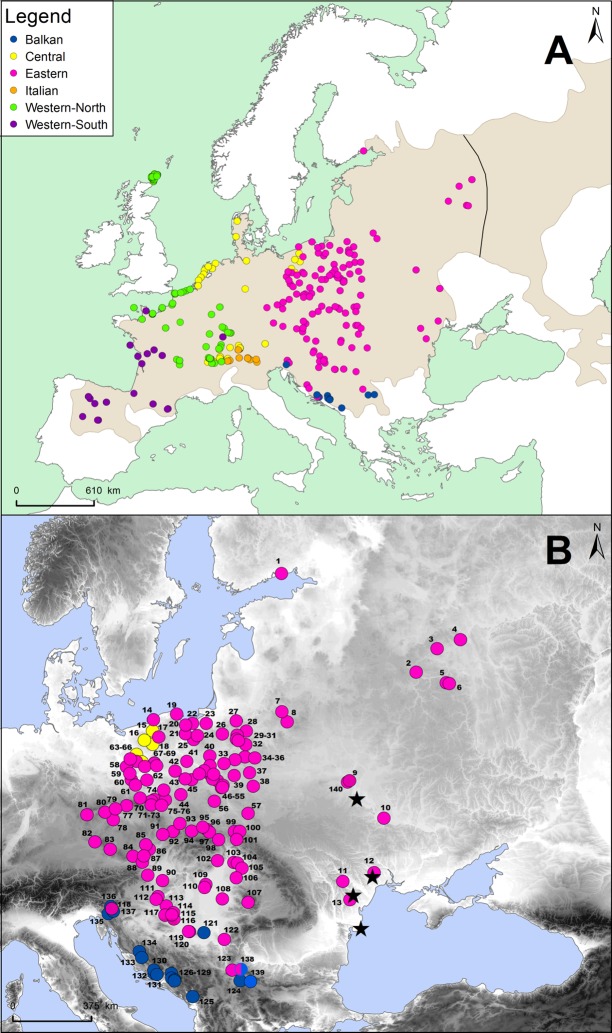
Map of distribution of mtDNA lineages of the common vole in Europe with new and previously obtained sequences. (A) Distribution of common vole cytochrome *b* samples used in this study, newly sequenced and from GenBank [24, 28–30]. The brown area shows the range of the species in Eurasia. The solid line marks the hybrid zone between the western (*arvalis*) and eastern (*obscurus*) forms; only the data for the western form are used in this study. Sampling localities are colored according to the cytochrome *b* lineage. (B) Distribution of common vole cytochrome *b* samples in central Europe belonging to the Eastern mtDNA lineage, both newly sequenced and from GenBank. The blue circles match the location of the Balkan mtDNA lineage, which makes contact with the Eastern lineage to the south. The yellow circles show the occurrence of the Central mtDNA lineage to the north of the area occupied by the Eastern lineage, including where they make contact. The black stars mark the locations where *Microtus rossiaemeridionalis* was found among the samples sequenced (KX380179-KX380191). Location numbers on the map are listed in S1, S2 and S3 Tables.

The common vole *Microtus arvalis* (Pallas, 1778) is one of the best studied small mammal species in Europe, in terms of phylogeography and population genetics. The common vole comprises two parapatric chromosomal races which are often considered subspecies, or even species, with the names *M*. *arvalis* and *M*. *obscurus* (Eversmann, 1841) (Fig 1A). The two forms may be distinguished both from their karyotypes and mitochondrial cytochrome *b* (cyt*b*) variation [23–25]. In this study we focus on the nominate form, for which six mitochondrial DNA (mtDNA) lineages have been described, based on cyt*b* (Fig 1A). Previous studies on the phylogeography of this species suggest that mtDNA is an excellent marker for identification of its glacial refugia and study of its range expansion, as in the case of its sympatric congener, *M*. *agrestis* [26, 27]. Three of the mtDNA lineages probably originated from southern refugia (the Western-South lineage from the Iberian Peninsula, the Italian lineage and the Balkan lineage [24, 28, 29]) while three lineages probably originated in northern refugia (the Western-North lineage from a refugium located in the vicinity of central France, the Central lineage from a refugium close to the Alps and the Eastern lineage, which may have been derived from the vicinity of the Carpathian Mountains [28, 30, 31]).

Previous studies of the Eastern lineage have been geographically limited, confined mainly to Poland [24, 30]. Based on these studies, it has previously been inferred that this lineage originated in the Carpathians, because of its distribution and the presence of fossils in the region over the last 25 000 years [30, 32, 33]. Earlier microsatellite analyses revealed subdivision of the Eastern mtDNA lineage into two genetic groups [34]. Given the results of the earlier studies from Poland, placed in the wider context of what is known about the common vole, the Eastern lineage is an excellent model system for an in-depth study of a small mammal population which potentially derives from a northern glacial refugium. Here, we collected many more individuals from central and eastern Europe to increase the range and number of individuals sampled. We analyzed 318 individuals from 140 locations in 13 countries, allowing us to determine the entire range of the Eastern lineage, and to more accurately identify both its refugial history and the subsequent expansion and re-colonization pattern of the species in this part of Europe.

Specifically, this broad sampling allows us to consider the following questions relating to occupation of northern glacial refugia by the common vole: (i) Is the pattern of mitochondrial and microsatellite variation consistent with the Eastern lineage deriving from the Carpathian Basin, as opposed to a refugium or refugia located elsewhere? (ii) How does the timing of expansion of the Eastern lineage relate to events during the last glaciation, particularly the LGM? (iii) Is the differentiation that is evident in Poland, based on microsatellite variation, replicated at a wider scale in the Eastern lineage and what is the implication for the refugial population and its subsequent expansion? Overall, the level and density of sampling in the area surrounding this northern refugium provides an exceptionally detailed perspective on the relationship between genetic variation in a contemporary population and the refugium, or refugia, from which it originated.

## Materials and Methods

All capture and handling procedures of voles in Poland were approved by the Local Ethics Committee for Animal Experiments in Białystok (Decision no: 5/2013). Samples of voles from Croatia, Czech Republic, Hungary, Romania, Serbia and Slovenia were collected according to the Legislation of Nature Protection and Legislation of Animal Welfare valid in these countries and permission to the Slovenian Museum of Natural History given by the Veterinary Administration of the Republic of Slovenia. No additional permits from Ethics Committee were needed. All capture and handling procedures of the common voles from Russia and Ukraine were approved by the Animal Care and Use Committee of the A.N. Severtsov Institute of Ecology and Evolution of the Russian Academy of Sciences (official letter from the deputy director of the Institute, no: 3485/09). No additional permits were required. Permission from the landowners was obtained where necessary. Samples were not collected from protected locations and field studies did not involve endangered, threatened or protected species.

### Mitochondrial DNA

We obtained new mtDNA data from 177 samples of the common vole from Poland, Belarus, Russia, Moldova, Ukraine, Czech Republic, Hungary, Slovenia, Croatia, Romania, and Serbia (Fig 1, S1 Table). Samples were collected between 1995 and 2015 and preserved in 96–98% ethanol. Additionally, our collection included samples from central and southern Ukraine and Armenia but all of them appeared to be the southern vole (*Microtus rossiaemeridionalis*), a sibling species almost identical in morphology to the common vole [35]. We obtained 13 complete cyt*b* sequences from those samples and they were identified using BLAST (https://blast.ncbi.nlm.nih.gov/Blast.cgi). They were not included further in the analyses performed in this study. The sequences obtained were deposited in GenBank (accession numbers: KX380179-KX380191).

Total genomic DNA was extracted using Syngen Tissue DNA Mini Kit in accordance with the manufacturer’s instructions. The amplification and sequencing of the complete cyt*b* sequence (1143 bp) were conducted according to methods described by Wójcik et al. [10], using primers described by Stojak et al. [30], in two overlapping fragments, each 500–600 bp in length. We also included negative PCR controls without template DNA. Additionally, we used 609 sequences obtained from previous studies (Fig 1A; for the whole list of locations see S1, S2 and S4 Tables in [30]). In all, we analyzed cyt*b* sequences from 786 individuals (Fig 1A, S1 and S2 Tables).

The nucleotide sequences were aligned and manually checked in BioEdit 7.2.5 [36]. As in our previous study [30], the complete alignment was shortened to 1000 bp. The distribution of polymorphic nucleotide and amino acid sites, number of transversions and transitions, synonymous and non-synonymous changes and position of stop codons in cyt*b* sequences obtained from newly-collected individuals were determined in DnaSP 5.1 [37] using the method of Degli Esposti et al. [38].

A median-joining network was constructed for all 786 sequences in Network 4.6 [39], to establish the overall pattern of mitochondrial genetic variation in the species.

We used Bayesian genealogy sampling in BEAST 1.8 [40], with all 786 sequences, to estimate the time to most recent common ancestor (tMRCA) for each lineage. The analysis was carried out according to the procedure described by Stojak et al. [30], using the HKY+Γ substitution model. A strict molecular clock was compared with an uncorrelated lognormal relaxed molecular clock [41]. Two demographic models were tested: skyline and constant population size [42]. For model selection, path sampling and stepping-stone sampling [43] were used to estimate marginal likelihoods (MLEs) for each model, based on four independent MCMC chains, each comprising 1000 steps of 100 000 generations, following 10 million generations burn-in. In each case, sampling was considered sufficient, as the estimates from the two methods (path sampling and stepping-stone sampling) converged on similar values. The MLEs were then used to obtain the Bayes Factor for each comparison to determine the best-fitting model [44]. The previously estimated clock rate of 3.27 x 10^−7^ substitutions/site/year^-1^ was used to calibrate the genealogy and date tMRCAs of each lineage [28, 30]. Posterior distributions of tMRCA and other model parameters were obtained from four independent MCMC simulations (each run for 200 million generations, with effective sample size for each parameter over 200), using TRACER 1.5 [45]. Posterior samples from the four chains were combined, using LogCombiner, part of the BEAST package. The Maximum Clade Credibility (MCC) tree was obtained using TreeAnnotator, part of the BEAST package, and visualized with FigTree 1.4 (http://tree-bio.ed.ac.uk/). The analyses were repeated without sequence data to test the effect of the joint prior distributions on the posterior distributions for each parameter of interest.

Nucleotide and haplotype diversity were calculated in DnaSP. To test for recent population expansion in mitochondrial lineages, we applied three tests in Arlequin 3.5.1.2 [46]: Tajima’s [47] *D* and Fu’s [48] *F*_*S*_ statistics and the mismatch distribution using sum of squared deviations SSD [49], with significance inferred using 1000 bootstrap replicates. Subsequently, we calculated time since expansion for lineages in which all three tests confirmed recent expansion (Tajima’s *D* and Fu’s *F*_*S*_ significant, SSD not significant; see [16, 30, 50]). The estimate was calculated according to a method based on the Tau value (Tau = 2ut, where u is the mutation rate and t is the mean generation time [51, 52]) using an online tool described by Schenekar and Weiss [53] (mismatch calculator, kindly provided by Stephen Weiss). Tau values were calculated in Arlequin, the substitution rate of 3.27 x 10^-7^substitutions/site/year^-1^ was applied and the generation time was assumed to be one year [28, 30]. To examine the demographic expansion of the Eastern lineage, a Bayesian skyline plot was prepared in TRACER, according to the method described in [30]. In this analysis we used all 318 sequences belonging to this lineage.

Geographic variation in nucleotide diversity (π) for the Eastern lineage was mapped with interpolation using ArcGIS 10.3.1 Geostatistical Analyst (ArcGIS Desktop: Release 10.3 Redlands, CA: Environmental Systems Research Institute 2015). Populations with only one individual were excluded to avoid cases where π is equal to 0. Altogether we used 293 sequences from 74 populations. Interpolation was carried out using an Inverse Distance Weight model (IDW, power = 1, based on 12 neighbors) which uses a linear-weighted combination set of sample points, assigned as a function of the distance of an input point from the output cell location. This means that the greater the distance is, the less influence the input cell obtained has on the results.

### Microsatellite DNA

We used 311 new specimens for microsatellite analysis, obtained from the Carpathian Basin, Russia and the Balkans, together with 188 specimens which were already analyzed in the previous study of Stojak et al. [34]. We initially used the previous panel of 11 microsatellite loci as described by Stojak et al. [34], following the same protocols. However, this was reduced for this study by the exclusion of loci AV12, Moe5 and MSM2, due to excessive missing data or risk in identification of false alleles as a result of non-specific amplification. Negative PCR controls without template DNA were included for each set of samples and each multiplex. We repeated the PCR for 15% of samples to test for error. All genotypes were independently scored three times. Altogether, we conducted the study on 499 individuals from 61 localities (Fig 1B). Sample sizes for populations varied, ranging from 1–16 individuals. A complete list of populations, localities and number of individuals are given in S3 Table.

Only populations with five or more individuals were included in all population-level analyses. Observed (H_O_) and expected heterozygosity (H_E_) were calculated in Arlequin. FSTAT v. 2.9.3 [54] was used to determine deviations from Hardy-Weinberg Equilibrium (HWE), allelic richness (AR) and to test for linkage disequilibrium between all pairs of loci in each population (using 10,000 simulations). Null alleles were identified in Micro-Checker v. 2.2.3 [55]. Pairwise F_ST_ values [56] between populations within each species were estimated in FSTAT, determining significance using 10,000 permutations under sequential Bonferroni corrections [57]. The relationship between pairwise genetic (F_ST_) and geographic distances was tested using a Mantel test [58] in IBDWS v. 3.23 [59].

To establish the number of genetic clusters occurring across central Europe we initially used Bayesian analysis in STRUCTURE 2.3.4 [60]. Ten independent runs of 500,000 generations with 100,000 generations of burn-in were performed for each value of *K* (1–20) under the admixture model and with the assumption that allele frequencies among populations are correlated. The optimal number of clusters were identified using the Evanno method (Δ*K*; [61]) and mean posterior probability of the data (Ln(*K*); log probability). Calculations were conducted in STRUCTURE HARVESTER [62]. We assigned populations to a particular cluster according to assignment probability *q* ≥ 0.8. Populations with assignment probability of 0.2 < *q* < 0.8 were classified as ‘intermediate’ [34, 63]. We used Clumpp 1.1.2 [64] and Distruct 1.1 [65] to assemble 10 independent runs of best-fitting values of *K* and to generate graphical representations.

Additionally, we performed Spatial Principal Components Analysis (sPCA; [66]) in R studio [67], to describe genetic differentiation and identify potential genetic structure across all populations from central Europe. Within that package, a G test was used to check for global (overall) structure and an L test for local (smaller scale) structure. Both tests were conducted using 999 permutations. The algorithm used in sPCA is modified such that the principal axes maximize spatial autocorrelation instead of correlation. Unlike methods such as STRUCTURE, sPCA does not take into consideration Hardy-Weinberg Equilibrium (HWE) or the extent of linkage disequilibrium [66, 68].

Interpolation of allelic richness (AR) and expected heterozygosity (H_E_) were conducted for 42 populations to identify potential hotspots of genetic diversity to complement the nucleotide diversity analysis with cyt*b*. Only populations with 5 or more individuals were included. Two Russian populations were excluded from this analysis because they were geographically distant from all the other populations. Analyses were carried out using ArcGIS 10.3.1, adopting the same model as for cyt*b*.

## Results

### Mitochondrial cyt*b* sequences

Amplification of the complete cyt*b* sequence (1143 bp) was successful for all 177 new samples of the common vole (S1 Table). We found no contamination in negative controls. The distribution of polymorphic nucleotide sites showed that substitutions are mostly in the third codon position, with a higher ratio of transitions (106Ts:13Tv; S4 Table). Non-synonymous changes occurred at the first codon position more often than at the second. Substitutions were mostly in the third codon position, with a higher ratio of transitions and only one (final) stop codon was identified (nucleotide positions 1141–1143). We found a much higher number of synonymous than non-synonymous changes (99: 20). These results are in agreement with a pattern that has been previously described by Irwin et al. [69] in mammals (and other taxa).

The 177 new sequences had 126 polymorphic sites, 85 of which were phylogenetically informative, and 97 haplotypes were identified. All 786 sequences analyzed contained 267 polymorphic sites, 181 of which were phylogenetically informative, and 309 haplotypes were identified. No nuclear mitochondrial DNA segments (numts) [70, 71] were found. PCR amplifications always produced only one band and we found no ambiguities in the sequences obtained–there were no unexpected stop codons, deletions, insertions or changes in the reading frame; all sequences were in concordance with the rest of cyt*b* sequences of the common vole from GenBank.

**Fig 2:**
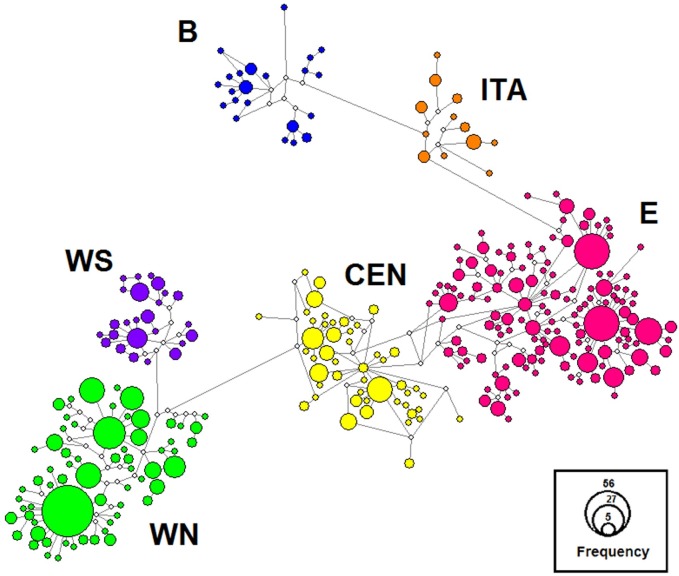
The network of cyt*b* haplotypes of the common vole from Europe. Median-joining network of cytochrome *b* haplotypes of the common vole, colored according to mtDNA lineage (blue–Balkan, B; orange–Italian, ITA; pink–Eastern, E; yellow–Central, CEN; violet–Western-South, WS; green–Western-North, WN). The area of the circles represents the number of sampled individuals with that haplotype. Small open circles indicate unsampled intermediate haplotypes.

The network analysis allowed us to assign the new sequences to the six previously described mtDNA lineages [24, 28–30, 72]. Eight new sequences from the Balkan region were assigned to the Balkan lineage, three from north-western Poland to the Central lineage and the remaining 166 to the Eastern lineage (Fig 2).

In the cyt*b* genealogy, illustrated by the MCC tree and obtained from MCMC simulations with a coalescent model, all of the sequences were again included in the six previously identified lineages (S1 Fig). Using all 786 sequences, the marginal likelihood estimates (MLEs) for skyline and constant population size demographic models were -9474.7 and -9684.5, respectively. A coalescent skyline model was therefore chosen over a constant population size model (Bayes Factors BF: 209.8) and an uncorrelated relaxed lognormal molecular clock was chosen over a strict molecular clock (MLEs: -9474.7 and -9509.4 respectively; BF: 34.7). In the Bayesian genealogy, all six lineages were highly supported (posterior probabilities either 0.99 or 1.0). While the Balkan and Italian lineages were entirely distinct from all of the other lineages, the Western-North and Western-South lineages formed a distinct and well-supported clade (PP = 0.95), as did the Central and Eastern lineages (PP = 0.99). The deeper splits in the genealogy were not supported and the relationship between the lineages is therefore uncertain.

The root of the genealogy was dated to 67.0 kya (with lower and upper 95% highest posterior density HPD limits of 41.8 and 104.5 kya, respectively;Table 1). The Eastern lineage, which is the lineage of main interest here, had a tMRCA estimate of 22.4 kya (95% HPD: 13.5–29.8 kya). The two Western lineages had similar tMRCA estimates of approximately 25 kya (95% HDP: 12.7–36.0 kya for Western-North and 12.0–37.5 kya for Western-South) (Table 1). The Balkan lineage had a tMRCA of 18.2 kya (95% HPD: 8.5–28.7 kya), whereas the tMRCAs of the Italian and Central lineages were 13.9 kya (95% HPD: 5.9–20.4 kya) and 16.3 kya (95% HPD: 8.5–22.6 kya), respectively (Table 1).

**Table 1:** Time to most recent common ancestor (tMRCA) for the common vole population and for the six cytochrome *b* lineages, with median and 95% highest posterior density (HPD) limits estimated from 720 million post-burn-in generations obtained from four independent MCMC simulations in BEAST.

	95% HPD lower limit (kya)	Median (kya)	95% HPD upper limit (kya)
Tree root	41.823	66.968	104.554
Eastern	13.468	22.376	29.815
Central	8.506	16.274	22.571
Western-North	12.719	24.621	36.003
Western-South	12.026	24.937	37.459
Italian	5.855	13.879	20.449
Balkan	8.530	18.200	28.749

Genetic variation in the three lineages from eastern Europe, which are those of interest here, is summarized in Table 2. Nucleotide diversity (range: 0.0050–0.0073) and haplotype diversity (range: 0.960–0.977) were highest for the Eastern lineage and lowest for the Central lineage. The Central and Eastern lineages had significant values of Tajima’s *D* and Fu’s *F*_*S*_ and non-significant SSD values, all of which indicate recent demographic expansion. Based on the mismatch Tau values, the Central lineage began to expand 6.3 kya (95% CI: 3.7–10.7 kya) and the Eastern lineage 8.3 kya (95% CI: 4.8–13.9 kya). The Bayesian skyline plot obtained for the Eastern lineage suggested that population expansion started approximately 10 kya (S2 Fig).

**Table 2:** Genetic variability within cytochrome *b* lineages of *Microtus arvalis*. It contains individuals newly-sequenced for this study, including number of haplotypes, nucleotide and haplotype diversity, neutrality tests statistics, mismatch distribution represented by sum of squared deviations (SSD) and time since expansion calculated from Tau values for those lineages showing significant values for both Tajima’s *D* and Fu’s *F*_*S*_ and not significant for SSD (see text).

Lineage	*N*	Number of haplotypes	Nucleotide diversity (π)	Haplotype diversity (*h*)	Tajima’s *D*	Fu’s *F*_*S*_	SSD	Tau (95% CI)	Time in ky since expansion (95% CI)
Balkan	30	22	0.0067	0.970	-1.037	-25.155***	0.006	5.170 (2.637–10.393)	-
Central	113	48	0.0050	0.960	-2.206 *	-25.123***	0.003	4.144 (2.343–6.999)	6.337 (3.722–10.702)
Eastern	318	135	0.0073	0.977	-2.028*	-24.377***	0.001	5.416 (3.160–9.092)	8.282 (4.832–13.903)

*N–*number of cyt*b* sequences

*, *P*<0.05

***, *P*<0.001; for Tajima’s *D*, Fu’s *F*_*S*_ and SSD statistics.

**Fig 3:**
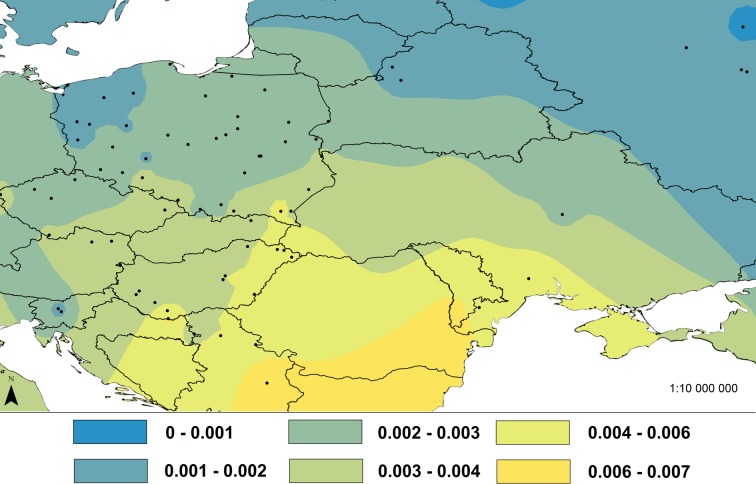
Interpolation of nucleotide diversity in common voles from central Europe. IDW interpolation of the nucleotide diversity (π) values of cytochrome *b* sequences from 74 populations of the common vole from the Eastern mtDNA lineage. The locations of populations are marked with black dots on the map and the π values for them are given in the text. The interpolated values of the nucleotide diversity based on 12 neighbors are presented in the map in different colors according to the legend. Only samples with ≥ 2 individuals were used for interpolation.

The interpolation of nucleotide diversity in the Eastern lineage showed that this is highest in the Carpathian area and decreases northwards with increasing distance from this area (Fig 3).

### Microsatellites

We analyzed eight microsatellite loci in 499 individuals of the common vole (S5 Table). There were only 0.33% missing data among all samples. No errors were found in the re-genotyped individuals. The estimates for H_O_ and H_E,_ together with the identification of null alleles, are shown in S6 Table. The diversity ranged from 0.535–0.903 (H_O_) and 0.581–0.925 (H_E_). Null alleles were identified only for Moe6 in a few populations. As this locus had little influence on the overall results, it was not excluded from further analyses (see below).

At the population level, values of allelic richness (AR), observed heterozygosity (H_O_) and expected heterozygosity (H_E_) varied from 4.49–7.47, 0.667–0.925 and 0.768–0.916, respectively (S3 Table). Only five populations significantly differed from HWE expectations after Bonferroni correction. Tests of linkage disequilibrium over all populations revealed significant linkage at all loci for two populations from Aleksinac (Serbia) and Česky Dub (Czech Republic).

Overall population differentiation was rather low (F_ST_ = 0.040, 95% CI: 0.032–0.048) and this value is nearly identical after removing the Moe6 locus (F_ST_ = 0.041, 95% CI: 0.031–0.050). There was significant differentiation between some pairs of populations (S7 Table). A Mantel test of the relationship between genetic and geographic distances revealed a weak and non-significant isolation by distance pattern for the common vole in central and eastern Europe *(r =* 0.1725, P = 0.068). This overall pattern of genetic differentiation is consistent with that from earlier studies of the common vole [31, 34].

**Fig 4:**
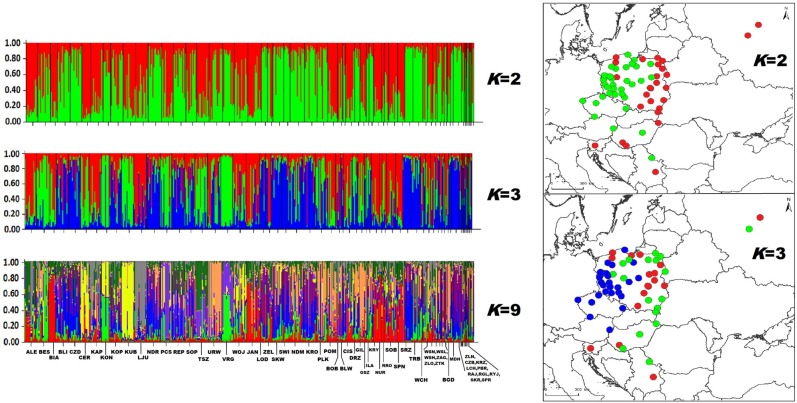
Bayesian clustering in STRUCTURE for the common vole from central Europe. Results of the Bayesian clustering analysis implemented in STRUCTURE under the admixture model, based on the microsatellite dataset. Plots: vertical columns represent the assignment probabilities to each of the inferred clusters identified for *K* = 2, 3 and 9. Numbers on horizontal axis match population localities shown in S3 Table. Maps: simplified representation of the structure revealed for *K* = 2 (above) and *K* = 3 (below). Localities are colored according to cluster with the highest assignment following the same coloring as the plots.

The values of Ln(*K*) and Δ*K*, obtained with STRUCTURE were not entirely consistent (S3 Fig). From Ln(*K*) there was support for *K* = 9, while from Δ*K* three values were supported (*K* = 2, 3 and 9). With *K* of two or three, there was a clear pattern of group membership within populations and this was reflected in the geographical distribution of the populations (Fig 4 and S4 Fig). In contrast, with *K* = 9 there was little consistency of group membership within populations (Fig 4).

In the sPCA analysis, only the highest eigenvalue is dominant (S5A and S5B Fig). The presence of positive eigenvalues indicates potential global patterns, however the G test revealed no significant global structure (observation = 0.0049, *P* = 0.225, S5C Fig). The L test results were also not significant, showing no local structure (observation = 0.0056, *P* = 0.225, S5D Fig). The plots with spatial representation of values for each population revealed that genetic differentiation between them was rather low and no distinct separation was observed (S6 Fig).

**Fig 5:**
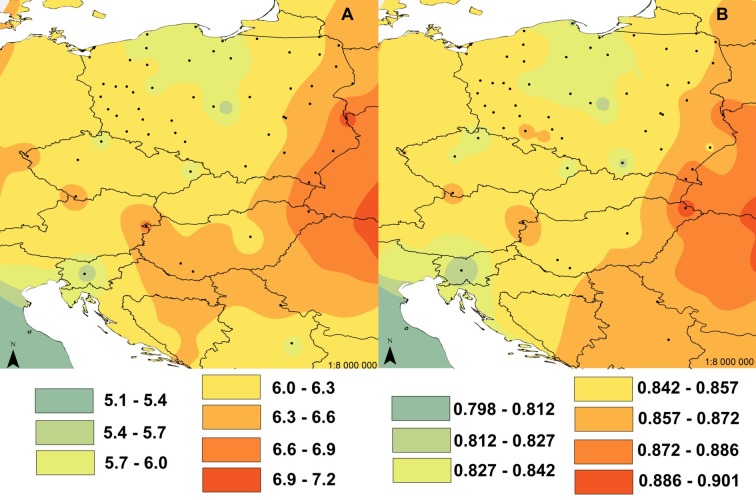
Interpolation of allelic richness and expected heterozygosity in common voles from central Europe. IDW interpolation of (A) allelic richness (AR) and (B) expected heterozygosity (H_E_) for microsatellites from 42 populations of the common vole in central Europe. The locations of populations are marked by black dots in the maps and the AR and H_E_ values for them are given in the text. The interpolated values of both indices based on 12 neighbors are presented in the maps in different colors according to the legend. Only samples with ≥ 5 individuals were used for interpolation. Two Russian populations were excluded from the analysis because they were so distant from other populations.

The interpolations of both allelic richness (AR, Fig 5A) and expected heterozygosity (H_E,_ Fig 5B) showed that diversity is highest in the north-eastern part of the Carpathian Basin and this diversity decreases gradually away from this area.

## Discussion

The common vole *Microtus arvalis* is a particularly well-studied phylogeographic model species in Europe. A number of previous studies have revealed multiple mtDNA lineages of this species, based on cyt*b* sequences, and the distribution of these lineages has been related to end-glacial biogeographic history [24, 28–30, 72]. The use of other genetic markers, such as microsatellites or those on the Y-chromosome, have generally revealed patterns congruent to those found with mtDNA [28, 31, 73, 74].

Here, we substantially enhanced the geographic coverage of cyt*b* sequences of *M*. *arvalis* in central and eastern Europe. In our analysis of cyt*b*, we made use of all 786 sequences available (including the samples in this paper and in GenBank) to confirm the six mtDNA lineages previously described in the species (Western-South, Western-North, Italian, Central, Eastern and Balkan; Fig 1A). Our material also included samples from central and southern Ukraine and Armenia but all of them appeared to be from the southern vole (*Microtus rossiaemeridionalis*), a sibling species almost identical in morphology to the common vole [35], suggesting that the range of the common vole does not extend into these areas.

While European temperate species were traditionally thought to have occupied Mediterranean glacial refugia, the possibility of more northerly refugia has been promoted since the 1980s [6, 9, 11–13, 16], however studies of present-day populations in the vicinity of these refugia and expansion routes from them have not been exceptionally detailed. The common vole lineages which may derive from such northern refugia are the Western-North lineage (with a refugium located somewhere in central France; [28]), the Central lineage (with a refugium located somewhere in the Alpine region; [73]) and the Eastern lineage (with a refugium located in the Carpathians; [30]). However, the status of any of these putative common vole refugia, as a source for contemporary genetic variation, has not been thoroughly examined. The Eastern lineage and the status of its proposed Carpathian refugium are the focus of the present study. The existence of northern refugia is very interesting in a physiological sense, as the survival of a temperate species in these refugia would suggest that it has high resistance to the severe, dry and sub-arctic climatic conditions present at such latitudes during the LGM and Younger Dryas [4, 5, 18, 21, 22]. Such populations may have been able to expand rapidly on amelioration of the climate after the end of the cold period. Northern refugia may therefore have made a substantial contribution to the contemporary genetic diversity of high latitudes in Europe [9, 13, 14].

We analyzed 318 cyt*b* sequences from the Eastern lineage, with excellent coverage of its distribution between the Balkans and Baltic Sea. This area which would have been affected by severe glacial conditions during the LGM [4, 5], but nevertheless includes the vicinity of the Carpathian Mountains, proposed as a refugium for a variety of animals and plants, including hornbeam (*Carpinus betulus*; [13]), crested newt (*Triturus cristatus*; [75]) and brown bear (*Ursus arctos*; [76]). The Carpathian region has a well-established fossil history, including remains of common voles and other small mammals, from the time of the LGM [32, 33].

To address the first question of our study, we tested several properties of the Eastern lineage, to establish if the pattern of its mitochondrial variation is consistent with its derivation from a refugium located in the vicinity of the Carpathian Mountains. The distribution of the lineage clearly supports this hypothesis, as it is present in a wide area of central Europe, with the Carpathians in the centre of its range. Moreover, we observed a continuous spread of the lineage north of the Carpathians. Three diversity indices also provide evidence in support of the hypothesis (Figs 3 and 5). Relatively high levels of genetic diversity are expected in refugial areas for temperate species, due to the survival of large, demographically stable populations over a long period of past climatic oscillations [77, 78]. Here we show, using both mtDNA (nucleotide diversity) and microsatellite markers (allelic richness and expected heterozygosity) that there are hotspots of genetic diversity for the common vole in the Carpathians and their close proximity. The interpolations and plots showed a distinct reduction of genetic diversity with distance from the Carpathian Basin and are also consistent with a northward route of re-colonization from there (Figs 3 and 5). Another, previously proposed, refugial area for this lineage is the Balkan Peninsula [24], but this seems unlikely as this area is occupied by another mtDNA lineage [29]. Although there are fossil records of the common vole from the vicinity of the Carpathians at the time of the LGM [32, 33], ancient bone fragments without associated DNA cannot give an indication of which lineage was present at that time [79–81]. A very similar pattern of contemporary DNA variation to that of the common vole is observed in the bank vole where the ‘Carpathian’ lineage is more widespread in central Europe than the ‘Balkan’ lineage, which is present only in close proximity to its refugial area [9]. In the case of the bank vole, gene flow and demographic expansion were also observed from the Carpathian region to the south, not only northwards. This pattern was inferred using an isolation by migration (IM) model, which employs a Bayesian coalescent method and relies on the assumption that there is continuing gene flow between the two descendant populations which were derived from the split of the ancestral population. The estimated rate of gene flow into the ‘Carpathian’ lineage was equal to zero, while the gene flow outward from this lineage was significant [9].

It may be also hypothesized that the Eastern lineage of the common vole derived from refugia located somewhere close to the Ural Mountains or the Russian Plains and spread from such distant areas to central Europe after the LGM. For instance, Deffontaine et al. [82] suggested that the Eastern clade of the bank vole most likely derived from a refugium in the vicinity of the Urals and expanded its range to central Europe. However, the range of the Eastern lineage of the common vole does not include areas to the east of the Vladimir region, where the *obscurus* chromosome race occurs. Therefore, this hypothesis does not seem to be supported.

The second purpose of our study was to establish the timing of expansion of the Eastern lineage and to determine how it relates to events which occurred during the last glaciation. Coalescent genealogy sampling in BEAST, using the molecular clock rate that was established from common vole ancient DNA [28], inferred a median tMRCA for the Eastern lineage of 22.4 kya (95% HPD limits of 13.5 and 29.8 kya). This indicates that the Eastern lineage originated from a genetic bottleneck around the time of the LGM, when the species was known to be present in the Carpathians [32, 33]. Based on the mismatch Tau value, the median time for the onset of expansion of the Eastern lineage was 8.3 kya (95% CI from 4.8 to 13.9 kya). This date is broadly consistent with the time of demographic expansion that was estimated from coalescent genealogy sampling, in the present and earlier studies, at around 10 kya (S2 Fig, also see Fig 4 in [30]). These various estimates suggest that the Eastern lineage of the common vole was present in the Carpathian Basin during both the LGM and YD periods and re-colonized central and eastern Europe from this refugium at the beginning of the Holocene, after the last glacial retreat.

The final purpose of our study was to determine if the microsatellite differentiation that we found in our previous study of genetic variation in common voles from Poland [34] would be confirmed in a broader area of central Europe and, if so, what information it reveals about the population history of the species in the region. The previous study [34] described an eastern-western subdivision in Poland for both *M*. *arvalis* and *M*. *agrestis*. In this study, STRUCTURE analysis with samples of *M*. *arvalis* suggested that this pattern is also evident on a wider scale in central Europe. The eastern group occurs across eastern Poland, eastern Hungary, Russia and Serbia and the western group is present in western Poland, western Hungary and the Czech Republic (Fig 4 and S4 Fig). Again, as in the previous study, there were mixed (presumably hybrid) populations with intermediate levels of assignation to one of the two genetic groups. These populations are located between the eastern and western groups, in a broad contact zone (see Fig 4 and S4 Fig). However, there is a need to be cautious with the STRUCTURE results, because three possible values of *K* or genetic clusters (*K* = 2, 3 or 9) were inferred. The choice of *K* = 2 is based on the *a priori* data from [34], and cannot therefore be viewed as definitive. Results from a second spatial genetics program, sPCA, showed very weak differentiation between all populations across central Europe. The sPCA revealed no genetic structure and no division into genetic groups was indicated (see S6 Fig). These results were supported by low overall differentiation (F_ST_ = 0.04) and significant differentiation between only some pairs of populations. There was a similar lack of confirmation of the STRUCTURE results in the previous study within Poland [34], where STRUCTURE revealed two genetic groups in Poland but Discriminant Analysis of Principal Components (DAPC) showed weak differentiation across the country.

This study is the first to examine genetic variation, using microsatellite data, in common vole populations from a wide area of central Europe. Previous studies were confined to western Europe [28, 31]. The samples used in these microsatellite analyses were collected from an area where the Eastern mtDNA lineage predominates, and only five populations from north-western Poland were assigned to the Central mtDNA lineage. As in our earlier study [34], we did not find congruence between the mtDNA and microsatellite results, as the few Central lineage populations were not distinctive from the Eastern lineage. This differs from what has been found in Switzerland, where microsatellites also show differentiation in the contact zone of the Central and Western mtDNA lineages of common vole [31, 73, 74]. The number of loci used in the different studies is similar: eight loci in this study, 11 loci in [34] and 12 loci in the study in Switzerland [31]. Given that relatively few samples that were not from the Eastern lineage were examined in our study, it is unwise to use our data to make strong statements about differentiation between lineages. However, we are in a position to discuss the degree of substructure within the Eastern lineage.

We assume that the weak eastern-western effect may reflect historical or ecological factors that influenced the common vole in this region. Division into two genetic groups may have resulted from environmental factors during the late Pleistocene and early Holocene. During the YD river corridors were very broad and covered by permafrost patches [83] and probably the Vistula river was a geographic barrier hindering gene flow between vole populations during that time. Around 10 kya, during the period of rapid warming at the beginning of the Holocene, deciduous forest expanded and the Polish territory became influenced by oceanic climate [84]. This resulted in flooding over a large area, which could have formed a barrier to re-colonization in the common vole. The middle Vistula river valley, built of sand dunes, was mostly reshaped by wind between 10.7 and 10.5 kya but its stabilization continued until 9.3 kya. In the case of the Hungarian Plain this process took even longer [84]. The *K* = 2 pattern on the map (Fig 4) seems to correspond quite well to the Vistula and Danube river valleys. The pattern observed for *K* = 3 has similarities to that obtained for *K* = 2. In that case the western group still has the same distribution while the eastern group shows a degree of subdivision. According to the conclusions presented above and our previous study [34], we assume that the pattern obtained for *K* = 2 may best explain the observed diversity in the common vole populations in central Europe. In either case, the pattern has become largely obscured by later gene flow, which explains the lack of structure that was recovered with the sPCA.

Extensive sampling allowed us to perform an in-depth analysis of genetic diversity throughout the Eastern lineage of the common vole. Results of our study suggest that this lineage expanded from the Carpathian refugium and support an assumption that the Carpathians play an important role as a biodiversity hotspot (see in [85] for a recent review). Our study also supports a hypothesis that a part of central and eastern Europe (present-day Poland) does indeed form a phylogeographic suture zone [10]. Other species display a contact zone in Poland, for example, the bank vole, the common shrew (*Sorex araneus*) and the weasel [10, 16, 86]. Phylogeographic investigations in the central and eastern parts of Europe are important to examine colonization processes, including the meeting of lineages and the relative impact of southern and northern refugia on the postglacial colonization history of Europe.

## Supporting Information

S1 Fig Bayesian genealogy of the common vole from Europe. Maximum clade credibility tree for 786 cytochrome *b* sequences of *Microtus arvalis*, summarized and annotated from 7200 trees re-sampled from 720 million post-burnin generations of Bayesian genealogy sampling. For genealogy calibration the substitution rate of 3.27 x 10^−7^ substitutions/site/year was used (see text). The horizontal axis is in thousands of years ago (kya). Posterior probabilities of basal nodes indicate support (≥ 0.95) for each of the six mtDNA lineages (B, Balkan; WS, Western-South; WN, Western-North; ITA, Italian; E, Eastern; CEN, Central) and for higher level lineages. Grey bars show 95% HPD intervals for time to most recent common ancestor of each lineage.
(TIF)

S2 Fig Bayesian skyline plot for the Eastern mtDNA lineage of the common vole. Bayesian skyline plot presenting demographic change in the Eastern mtDNA lineage of the common vole with the effective female population size on a log scale against time from the present to 17.5 kya.
(TIF)

S3 Fig The evaluation of the STRUCTURE results for the common vole from central Europe. The Evanno et al. [61] Δ*K* (continuous line, left Y axis) and the mean log probability Ln(*K*) (open points, right Y axis) results from STRUCTURE for *Microtus arvalis* from central Europe based on the microsatellite data.
(TIF)

S4 Fig Genetic structure in common voles from central Europe. Genetic structure of *Microtus arvalis* populations in central Europe, based on a Bayesian analysis of microsatellite data with *K* = 2. The pattern is consistent with the presence of western and eastern groups (as previously observed in [34]). Dots show point locations depicted according to the population assignment into one of two genetic clusters at *q* ≥ 0.8; white—western group, black—eastern group, shades of grey intermediate representation between the two genetic clusters (0.2 <*q*< 0.8, according to the legend). Locations on the map match localities given in Fig 1 and S3 Table.
(TIF)

S5 Fig The results of the sPCA for the common vole from central Europe. Spatial Principal Component Analysis (sPCA) based on microsatellites of the common vole from central Europe. (A) Positive and negative eigenvalues which reflect potential global and local structure respectively. Only the first principal component is of sufficient magnitude to be retained. (B) Distribution of each eigenvalue according to its spatial autocorrelation and variance. ʎ1 represents the first, highest positive eigenvalue, marked in red in part A. (C) Results of a G test which tests the whole dataset in searching for global structure and (D) an L test for specifying possible local structure. The grey bars indicate the simulated values and the black diamond indicate the actual observed values. In neither case are the observed values outside the distribution of the simulated values and therefore there is no significant global or local structure.
(TIF)

S6 Fig The sPCA plots for the common vole from central Europe. Two representations of scores obtained for each population using Spatial Principal Components Analysis (sPCA), with a grid indicating the geographical relationship of the populations. The black and white squares (or grey level variant) represent positive and negative values respectively. Only populations with ≥ 5 individuals sampled were included. (A) Size of the squares reflects their differentiation: small squares are less differentiated than large ones. (B) Different absolute values are presented in different shades of grey. Neither plot shows a distinct pattern of genetic structure among the populations.
(TIF)

S1 Table List of specimens of *Microtus arvalis* for which new cytochrome *b* sequences were obtained for this study, including their site of origin (country codes: PL–Poland, BY–Belarus, RU–Russia, MDV–Moldova, UKR–Ukraine, RO–Romania, SLO–Slovenia, CR–Croatia, CZ–Czech Republic, HU–Hungary, SB–Serbia). (DOCX)

S2 Table List of those collection localities from central and eastern Europe mapped in Fig 1B which provided previously published data for this study but which were not used to generate new cyt*b* or microsatellite data. The full list of previously published cyt*b* sequences that were used in this study are available in S1, S2 and S4 Tables in [30]. The numbers in square brackets are consistent with the References section. The reference not used in the References section is marked by ‘*’ and its full description is given below the table.
(DOCX)

S3 Table Diversity indices for samples of *Microtus arvalis* used in this study for microsatellite analysis including sampling locality with geographic coordinates, sample size (n), observed (H_O_) and expected (H_E_) heterozygosity, allelic richness (AR) and the inbreeding coefficient (F_IS_). Samples used in the previous study of Stojak et al. [34] are marked by ‘#’. Significant values for F_IS_ are given after Bonferroni correction and marked by ‘*’. Only samples with ≥ 5 individuals were used for calculations.
(DOCX)

S4 Table Distribution of nucleotide polymorphisms with data organized by individual nucleotides (upper panel) and triplets (lower panel) in the *Microtus arvalis* cytochrome *b* sequences from newly-collected samples from central Europe. (DOCX)

S5 Table The microsatellite data obtained in this study for 8 loci of the common vole. The missing data are marked by ‘-9’.
(XLSX)

S6 Table Characteristics of loci used in the microsatellite analysis of the whole *Microtus arvalis* dataset including observed (H_O_) and expected (H_E_) heterozygosity and null alleles. (DOCX)

S7 Table Estimates of multilocus FST between all population pairs of *Microtus arvalis* based on microsatellites. Population labels are given according to S3 Table. Significant values are given after Bonferroni correction and marked by ‘*’.
(DOCX)
