# Supplementary figures and images for: Between the Balkans and the Baltic: Phylogeography of a Common Vole Mitochondrial DNA Lineage Limited to Central Europe
Source: PLoS One. 2016 Dec 16;11(12):e0168621. doi: 10.1371/journal.pone.0168621 (PMC5161492; doi:10.1371/journal.pone.0168621)

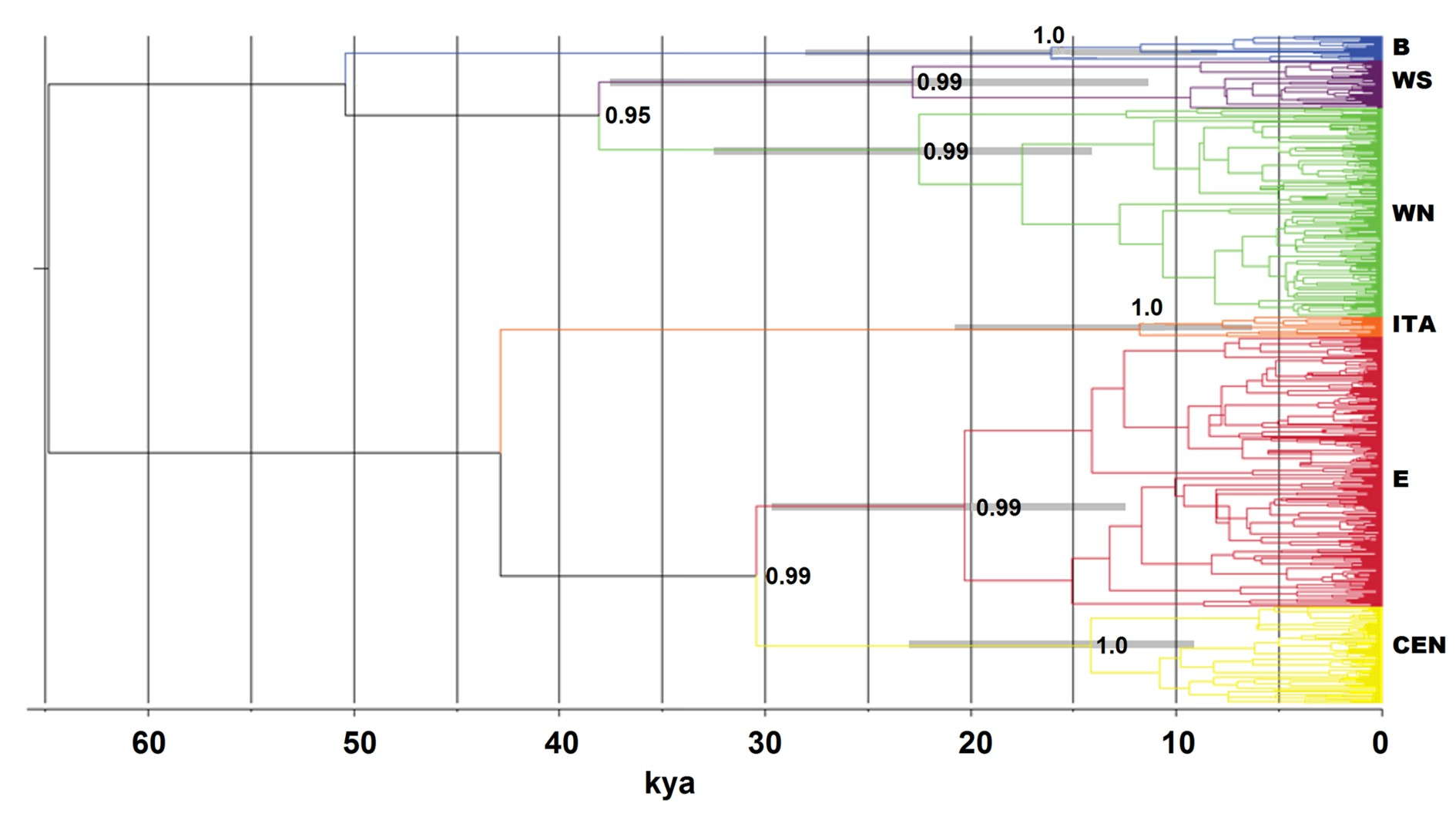

Supplement: S1 Fig — Maximum clade credibility tree for 786 cytochrome b sequences of Microtus arvalis, summarized and annotated from 7200 trees re-sampled from 720 million post-burnin generations of Bayesian genealogy sampling. For genealogy calibration the substitution rate of 3.27 x 10^−7^ substitutions/site/year was used (see text). The horizontal axis is in thousands of years ago (kya). Posterior probabilities of basal nodes indicate support (≥ 0.95) for each of the six mtDNA lineages (B, Balkan; WS, Western-South; WN, Western-North; ITA, Italian; E, Eastern; CEN, Central) and for higher level lineages. Grey bars show 95% HPD intervals for time to most recent common ancestor of each lineage. (TIF) [file pone.0168621.s001.tif]

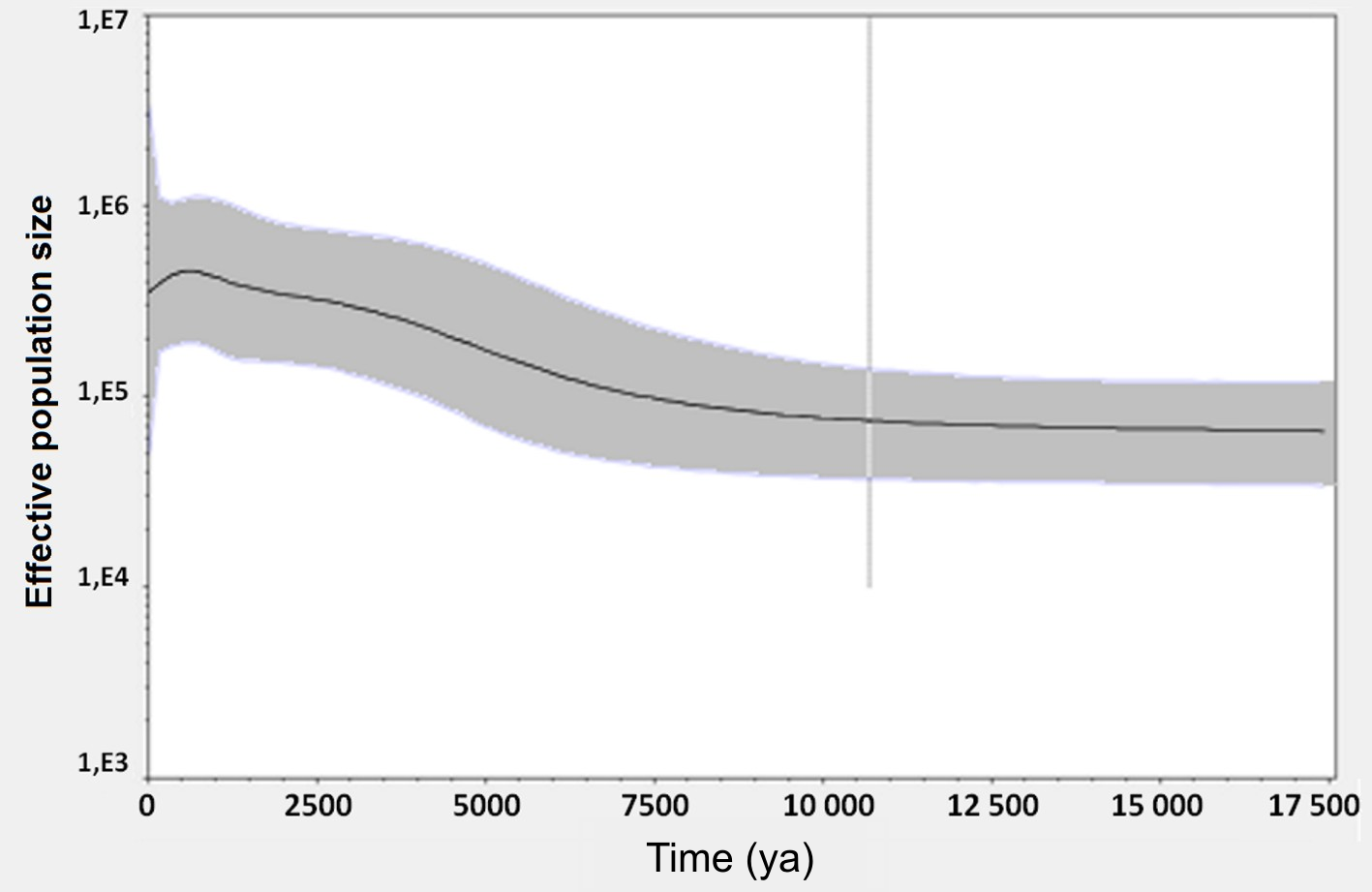

Supplement: S2 Fig — Bayesian skyline plot presenting demographic change in the Eastern mtDNA lineage of the common vole with the effective female population size on a log scale against time from the present to 17.5 kya. (TIF) [file pone.0168621.s002.tif]

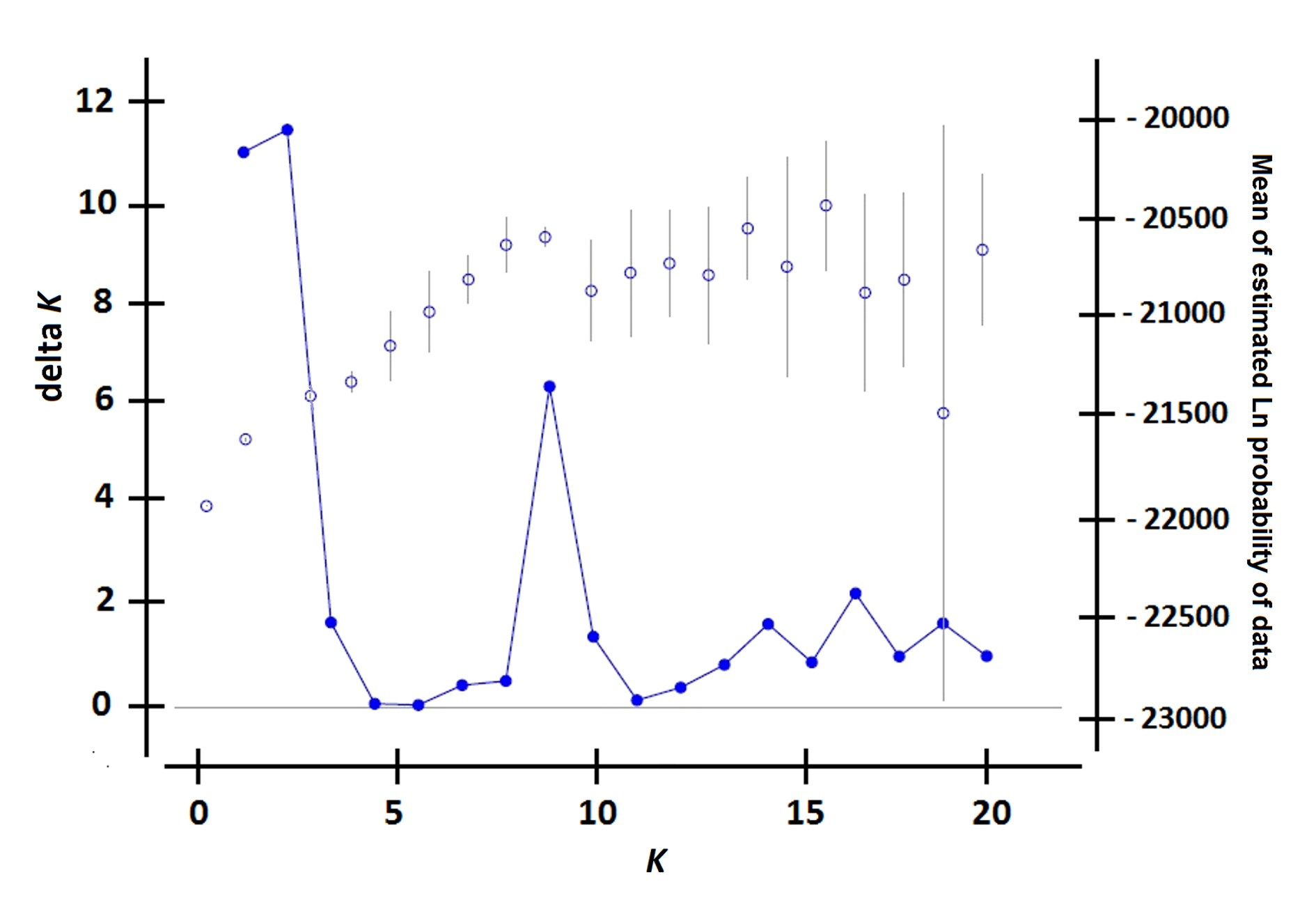

Supplement: S3 Fig — The Evanno et al. [61] ΔK (continuous line, left Y axis) and the mean log probability Ln(K) (open points, right Y axis) results from STRUCTURE for Microtus arvalis from central Europe based on the microsatellite data. (TIF) [file pone.0168621.s003.tif]

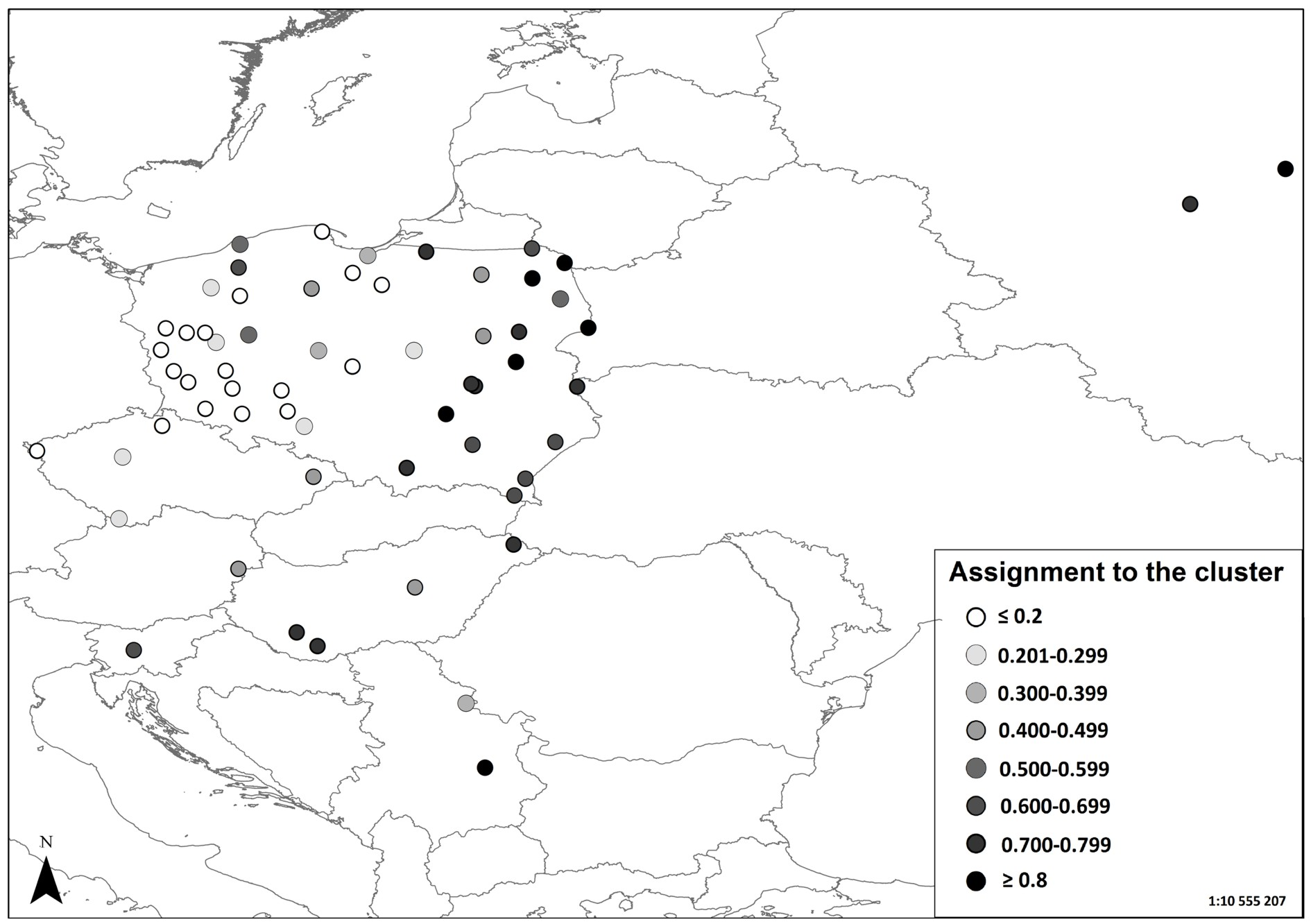

Supplement: S4 Fig — Genetic structure of Microtus arvalis populations in central Europe, based on a Bayesian analysis of microsatellite data with K = 2. The pattern is consistent with the presence of western and eastern groups (as previously observed in [34]). Dots show point locations depicted according to the population assignment into one of two genetic clusters at q ≥ 0.8; white—western group, black—eastern group, shades of grey intermediate representation between the two genetic clusters (0.2 <q< 0.8, according to the legend). Locations on the map match localities given in Fig 1 and S3 Table. (TIF) [file pone.0168621.s004.tif]

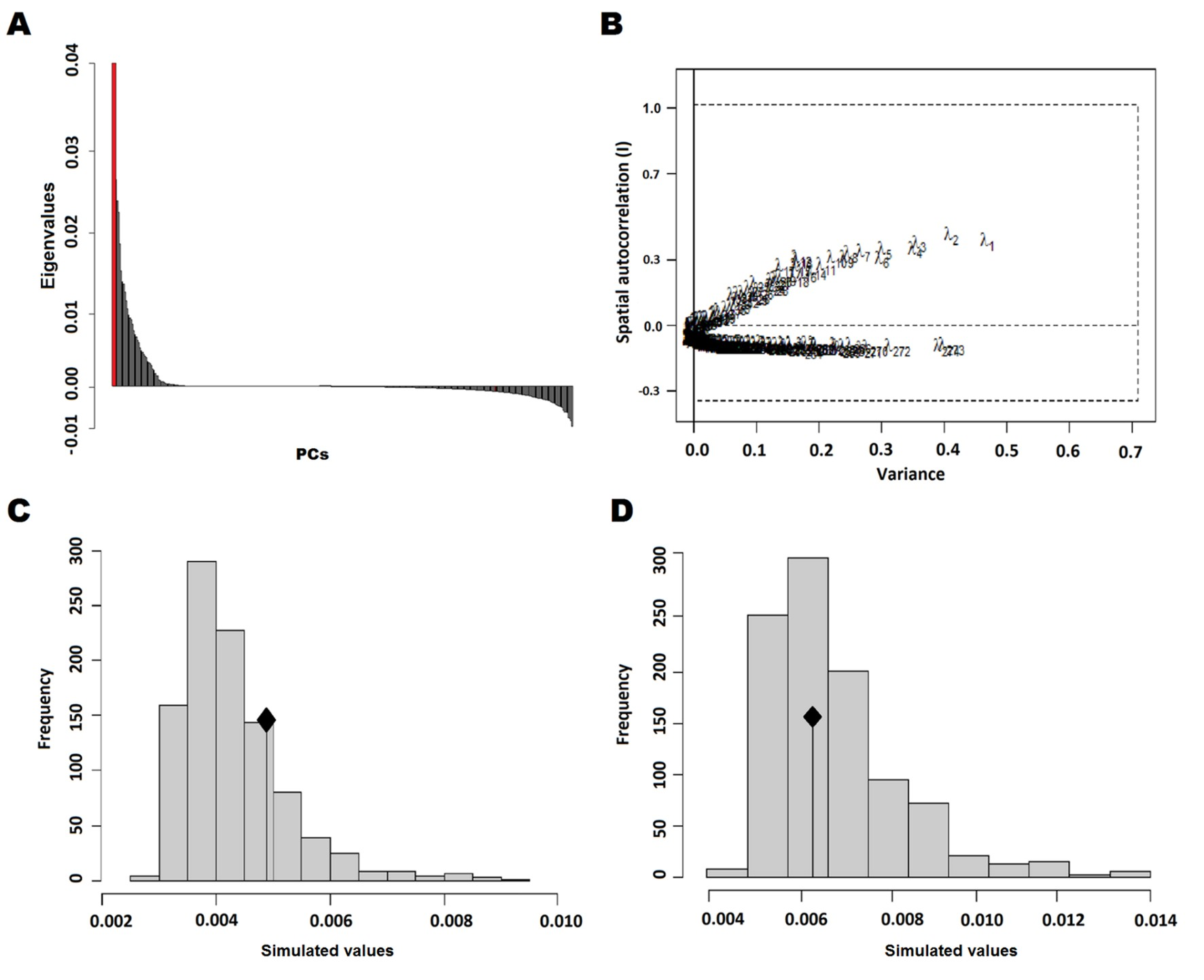

Supplement: S5 Fig — Spatial Principal Component Analysis (sPCA) based on microsatellites of the common vole from central Europe. (A) Positive and negative eigenvalues which reflect potential global and local structure respectively. Only the first principal component is of sufficient magnitude to be retained. (B) Distribution of each eigenvalue according to its spatial autocorrelation and variance. ʎ1 represents the first, highest positive eigenvalue, marked in red in part A. (C) Results of a G test which tests the whole dataset in searching for global structure and (D) an L test for specifying possible local structure. The grey bars indicate the simulated values and the black diamond indicate the actual observed values. In neither case are the observed values outside the distribution of the simulated values and therefore there is no significant global or local structure. (TIF) [file pone.0168621.s005.tif]

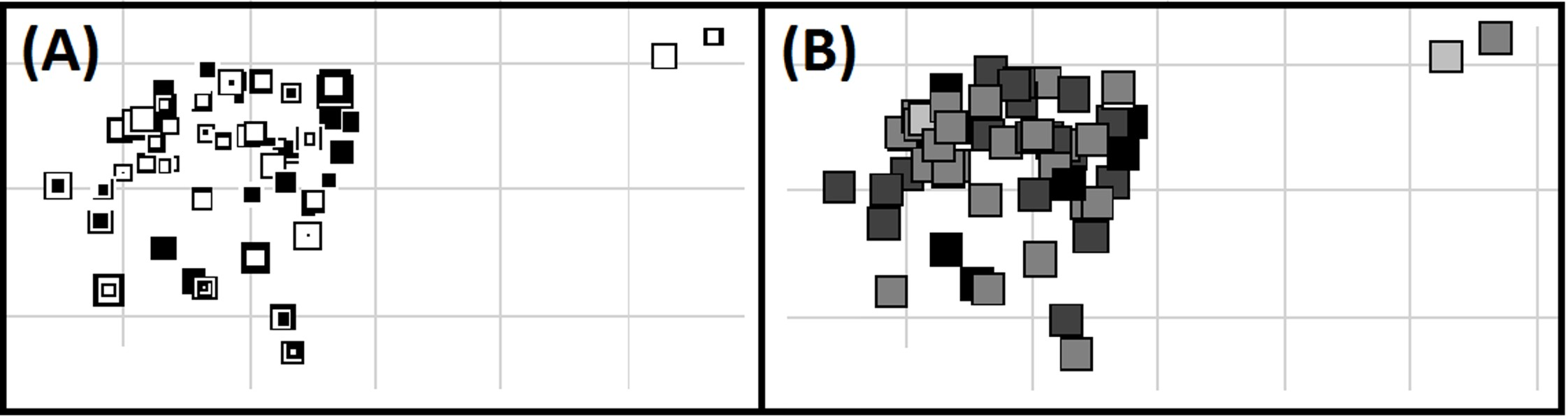

Supplement: S6 Fig — Two representations of scores obtained for each population using Spatial Principal Components Analysis (sPCA), with a grid indicating the geographical relationship of the populations. The black and white squares (or grey level variant) represent positive and negative values respectively. Only populations with ≥ 5 individuals sampled were included. (A) Size of the squares reflects their differentiation: small squares are less differentiated than large ones. (B) Different absolute values are presented in different shades of grey. Neither plot shows a distinct pattern of genetic structure among the populations. (TIF) [file pone.0168621.s006.tif]
