# Supplementary material for: Between the Balkans and the Baltic: Phylogeography of a Common Vole Mitochondrial DNA Lineage Limited to Central Europe
Source: PLoS One. 2016 Dec 16;11(12):e0168621. doi: 10.1371/journal.pone.0168621 (PMC5161492; doi:10.1371/journal.pone.0168621)
Supplement: S1 Table — (DOCX) [file pone.0168621.s007.docx]

**S1 Table. List of specimens of *Microtus arvalis* for which new cytochrome *b* sequences were obtained for this study, including their site of origin** (country codes: PL – Poland, BY – Belarus, RU – Russia, MDV – Moldova, UKR – Ukraine, RO – Romania, SLO – Slovenia, CR – Croatia, CZ – Czech Republic, HU – Hungary, SB – Serbia).

| **Tissue number** | **DNAID number** | **Location** | **Latitude (N)** | **Longitude (E)** | **Map reference (see Fig 1B)** | **cyt *b* lineage** | **GenBank Accession Number** |
| --- | --- | --- | --- | --- | --- | --- | --- |
| JS033 | JS033 | Białowieża, PL | 52.70 | 23.87 | 36 | Eastern | KX380105 |
| JS034 | JS034 | Białowieża, PL | 52.70 | 23.87 | 36 | Eastern | KX380106 |
| JS035 | JS035 | Białowieża, PL | 52.70 | 23.87 | 36 | Eastern | KX380107 |
| JS071 | JS071 | BielskPodlaski, PL | 52.77 | 23.19 | 35 | Eastern | KX380119 |
| JS072 | JS072 | BielskPodlaski, PL | 52.77 | 23.19 | 35 | Eastern | KX380120 |
| JS120 | JS120 | Urwitałt, PL | 53.81 | 21.64 | 26 | Eastern | KX380147 |
| JS121 | JS121 | Urwitałt, PL | 53.81 | 21.64 | 26 | Eastern | KX380148 |
| JS122 | JS122 | Urwitałt, PL | 53.81 | 21.64 | 26 | Eastern | KX380149 |
| JS123 | JS123 | Urwitałt, PL | 53.81 | 21.64 | 26 | Eastern | KX380150 |
| JS053 | JS053 | Grodzisk Mazowiecki, PL | 52.10 | 20.64 | 48 | Eastern | KX380113 |
| JS043 | JS043 | Januszno, PL | 51.49 | 21.51 | 54 | Eastern | KX380110 |
| JS046 | JS046 | Januszno, PL | 51.49 | 21.51 | 54 | Eastern | KX380111 |
| JS048 | JS048 | NowyDwór Mazowiecki, PL | 52.45 | 20.69 | 40 | Eastern | KX380112 |
| JS041 | JS041 | Poddębice, PL | 51.89 | 18.96 | 46 | Eastern | KX380108 |
| JS042 | JS042 | Poddębice, PL | 51.89 | 18.96 | 46 | Eastern | KX380109 |
| JS064 | JS064 | Konin, PL | 52.22 | 18.25 | 42 | Eastern | KX380115 |
| JS065 | JS065 | Konin, PL | 52.22 | 18.25 | 42 | Eastern | KX380116 |
| JS066 | JS066 | Konin, PL | 52.22 | 18.25 | 42 | Eastern | KX380117 |
| JS068 | JS068 | Konin, PL | 52.22 | 18.25 | 42 | Eastern | KX380118 |
| JS059 | JS059 | Zielątkowo, PL | 52.55 | 16.80 | 68 | Eastern | KX380114 |
| JS077 | JS077 | Sanok, PL | 49.56 | 22.21 | 99 | Eastern | KX380123 |
| JS075 | JS075 | Wojtkówka, PL | 49.56 | 22.56 | 100 | Eastern | KX380121 |
| JS076 | JS076 | Wojtkówka, PL | 49.56 | 22.56 | 100 | Eastern | KX380122 |
| JS078 | JS078 | Wojtkówka, PL | 49.56 | 22.56 | 100 | Eastern | KX380124 |
| JS079 | JS079 | Wojtkówka, PL | 49.56 | 22.56 | 100 | Eastern | KX380125 |
| JS097 | JS097 | StaraWieś, PL | 49.68 | 20.40 | 95 | Eastern | KX380127 |
| JS096 | JS096 | Naszacowice, PL | 49.56 | 20.56 | 97 | Eastern | KX380126 |
| JS101 | JS101 | Owsianka, PL | 50.98 | 16.87 | 73 | Eastern | KX380131 |
| JS102 | JS102 | Owsianka, PL | 50.98 | 16.87 | 73 | Eastern | KX380132 |
| JS098 | JS098 | Strzelce, PL | 50.91 | 16.66 | 72 | Eastern | KX380128 |
| JS103 | JS103 | Strzelce , PL | 50.91 | 16.66 | 72 | Eastern | KX380133 |
| JS104 | JS104 | Strzelce , PL | 50.91 | 16.66 | 72 | Eastern | KX380134 |
| JS099 | JS099 | Dzierżoniów, PL | 50.73 | 16.66 | 71 | Eastern | KX380129 |
| JS100 | JS100 | Dzierżoniów, PL | 50.73 | 16.66 | 71 | Eastern | KX380130 |
| JS105 | JS105 | Bronowice , PL | 52.91 | 15.50 | 64 | Central | KX380135 |
| JS111 | JS111 | Bronowice , PL | 52.91 | 15.50 | 64 | Central | KX380141 |
| JS107 | JS107 | Skwierzyna, PL | 52.60 | 15.51 | 63 | Eastern | KX380137 |
| JS108 | JS108 | Skwierzyna, PL | 52.60 | 15.51 | 63 | Eastern | KX380138 |
| JS109 | JS109 | Skwierzyna, PL | 52.60 | 15.51 | 63 | Eastern | KX380139 |
| JS110 | JS110 | Skwierzyna, PL | 52.60 | 15.51 | 63 | Eastern | KX380140 |
| JS106 | JS106 | Trzebicz, PL | 52.81 | 15.75 | 65 | Eastern | KX380136 |
| JS112 | JS112 | Trzebicz, PL | 52.81 | 15.75 | 65 | Eastern | KX380142 |
| JS113 | JS113 | Trzebicz, PL | 52.81 | 15.75 | 65 | Central | KX380143 |
| JS117 | JS117 | Wyczechy, PL | 53.69 | 17.04 | 17 | Eastern | KX380144 |
| JS118 | JS118 | Wyczechy, PL | 53.69 | 17.04 | 17 | Eastern | KX380145 |
| JS119 | JS119 | Wyczechy, PL | 53.69 | 17.04 | 17 | Eastern | KX380146 |
| B_1232 | B_1232 | Wołożyn, BY | 54.14 | 26.41 | 8 | Eastern | KX380176 |
| B_1233 | B_1233 | Wołożyn, BY | 54.14 | 26.41 | 8 | Eastern | KX380177 |
| B_1234 | B_1234 | Wołożyn, BY | 54.14 | 26.41 | 8 | Eastern | KX380178 |
| B-20 | B-20 | Soly, BY | 54.60 | 26.11 | 7 | Eastern | KX380151 |
| B-21 | B-21 | Soly, BY | 54.60 | 26.11 | 7 | Eastern | KX380152 |
| G13_RU | G13_RU | Czernogolowka, RU | 56.01 | 38.39 | 3 | Eastern | KX380173 |
| G32_RU | G32_RU | Czernogolowka, RU | 56.01 | 38.39 | 3 | Eastern | KX380175 |
| G5_RU | G5_RU | Czernogolowka, RU | 56.01 | 38.39 | 3 | Eastern | KX380174 |
| G8_RU | G8_RU | Czernogolowka, RU | 56.01 | 38.39 | 3 | Eastern | KX380172 |
| 12-18RU | 12-18RU | Kubinka – Nara, RU | 55.28 | 36.40 | 2 | Eastern | KX380158 |
| 12-19RU | 12-19RU | Kubinka – Nara, RU | 55.28 | 36.40 | 2 | Eastern | KX380159 |
| 7RU | 7RU | Kubinka – Nara, RU | 55.28 | 36.40 | 2 | Eastern | KX380168 |
| 8RU | 8RU | Kubinka – Nara, RU | 55.28 | 36.40 | 2 | Eastern | KX380169 |
| MIS-1D5 | MIS-1D5 | Kubinka – Nara, RU | 55.28 | 36.40 | 2 | Eastern | KX380165 |
| 11-50RU | 11-50RU | Ozyory lakes, RU | 54.51 | 38.33 | 5 | Eastern | KX380155 |
| 11-58RU | 11-58RU | Ozyory lakes, RU | 54.51 | 38.33 | 5 | Eastern | KX380156 |
| 11-59RU | 11-59RU | Ozyory lakes, RU | 54.51 | 38.33 | 5 | Eastern | KX380157 |
| 13-22RU | 13-22RU | Ozyory lakes, RU | 54.51 | 38.33 | 5 | Eastern | KX380163 |
| 06-38 RU | 06-38 RU | Zaraysk, RU | 54.44 | 38.52 | 6 | Eastern | KX380166 |
| 06-39 RU | 06-39 RU | Zaraysk, RU | 54.44 | 38.52 | 6 | Eastern | KX380167 |
| K_RU | K_RU | Chisinau, MDV | 47.01 | 28.86 | 11 | Eastern | KX380153 |
| 29o_RU | 29o_RU | Tarutine, UKR | 46.18 | 29.15 | 13 | Eastern | KX380170 |
| 36o_RU | 36o_RU | Tarutine, UKR | 46.18 | 29.15 | 13 | Eastern | KX380160 |
| 37o_RU | 37o_RU | Tarutine, UKR | 46.18 | 29.15 | 13 | Eastern | KX380161 |
| 41o_RU | 41o_RU | Tarutine, UKR | 46.18 | 29.15 | 13 | Eastern | KX380171 |
| 27UKR_RU | 27UKR_RU | Tylihul Estuary, UKR | 47.20 | 30.88 | 12 | Eastern | KX380162 |
| 28UKR_RU | 28UKR_RU | Tylihul Estuary, UKR | 47.20 | 30.88 | 12 | Eastern | KX380164 |
| 8UKR_RU | 8UKR_RU | Tylihul Estuary, UKR | 47.20 | 30.88 | 12 | Eastern | KX380154 |
| 242/14BK | 748BK | Curtuiseni, RO | 47.55 | 22.20 | 106 | Eastern | KX380104 |
| 223/14BK | 751BK | Curtuiseni, RO | 47.56 | 22.21 | 106 | Eastern | KX380091 |
| 251/14BK | 740BK | Scarisoara, RO | 46.46 | 22.87 | 107 | Eastern | KX380093 |
| 229/08 | 413BK | Medvode, SLO | 46.14 | 14.41 | 135 | Eastern | KX380101 |
| 231/08-1 | 414BK | Medvode, SLO | 46.14 | 14.41 | 135 | Eastern | KX380102 |
| 232/08-1 | 415BK | Medvode, SLO | 46.14 | 14.41 | 135 | Balkan | KX380044 |
| TK485/11 | 321BK | Mohovo, CR | 45.25 | 19.22 | 120 | Eastern | KX380100 |
| TK488/11 | 322BK | Opatovac, CR | 45.26 | 19.17 | 119 | Eastern | KX380022 |
| JL-1417 | 627BK | Bližna, CZ | 48.72 | 14.10 | 82 | Eastern | KX380038 |
| JL-1418 | 628BK | Bližna, CZ | 48.72 | 14.10 | 82 | Eastern | KX380057 |
| JL-1419 | 626BK | Bližna, CZ | 48.72 | 14.10 | 82 | Eastern | KX380092 |
| JL-1962 | 629BK | Buzica, CZ | 48.32 | 21.04 | 102 | Eastern | KX380039 |
| JL-1969 | 650BK | Buzica, CZ | 48.32 | 21.04 | 102 | Eastern | KX380058 |
| JL-1970 | 652BK | Buzica, CZ | 48.32 | 21.04 | 102 | Eastern | KX380059 |
| JL-1972 | 648BK | Buzica, CZ | 48.33 | 21.05 | 102 | Eastern | KX380056 |
| JL-1 | 653BK | Česky Dub, CZ | 50.66 | 15.00 | 77 | Eastern | KX380040 |
| JL-3 | 655BK | Česky Dub, CZ | 50.66 | 15.00 | 77 | Eastern | KX380060 |
| JL-5 | 656BK | Česky Dub, CZ | 50.66 | 15.00 | 77 | Eastern | KX380061 |
| JL-614 | 692BK | Fladnice, CZ | 48.48 | 15.59 | 83 | Eastern | KX380041 |
| JL-615 | 693BK | Fladnice, CZ | 48.48 | 15.59 | 83 | Eastern | KX380042 |
| JL-616 | 694BK | Fladnice, CZ | 48.48 | 15.59 | 83 | Eastern | KX380066 |
| JL-617 | 695BK | Fladnice, CZ | 48.48 | 15.59 | 83 | Eastern | KX380020 |
| JL-618 | 696BK | Fladnice, CZ | 48.48 | 15.59 | 83 | Eastern | KX380043 |
| JL-645 | 720BK | Koprivnice, CZ | 49.60 | 18.14 | 92 | Eastern | KX380068 |
| JL-646 | 721BK | Koprivnice, CZ | 49.60 | 18.14 | 92 | Eastern | KX380069 |
| JL-642 | 717BK | Koprivnice, CZ | 49.60 | 18.14 | 92 | Eastern | KX380067 |
| JL-1202 | 614BK | Mikulov, CZ | 48.81 | 16.64 | 85 | Eastern | KX380037 |
| JL-173 | 666BK | NovyDrahov, CZ | 50.14 | 12.39 | 81 | Eastern | KX380062 |
| JL-174 | 668BK | NovyDrahov, CZ | 50.14 | 12.39 | 81 | Eastern | KX380064 |
| JL-175 | 669BK | NovyDrahov, CZ | 50.14 | 12.39 | 81 | Eastern | KX380063 |
| JL-176 | 670BK | NovyDrahov, CZ | 50.14 | 12.39 | 81 | Eastern | KX380065 |
| JL-2520 | 556BK | Reporyje, CZ | 50.01 | 14.17 | 78 | Eastern | KX380046 |
| JL-2522 | 558BK | Reporyje, CZ | 50.01 | 14.17 | 78 | Eastern | KX380103 |
| JL-2523 | 559BK | Reporyje, CZ | 50.01 | 14.17 | 78 | Eastern | KX380054 |
| JL-2519 | 555BK | Reporyje, CZ | 50.01 | 14.17 | 78 | Eastern | KX380036 |
| JL-346 | 585BK | Velemin, CZ | 50.32 | 13.58 | 80 | Eastern | KX380051 |
| JL-347 | 586BK | Velemin, CZ | 50.32 | 13.58 | 80 | Eastern | KX380052 |
| JL-349 | 588BK | Velemin, CZ | 50.32 | 13.58 | 80 | Eastern | KX380055 |
| JL-345 | 584BK | Velemin, CZ | 50.32 | 13.58 | 80 | Eastern | KX380047 |
| 268/10 | 314BK | Besenyszög, HU | 47.30 | 20.26 | 109 | Eastern | KX380081 |
| 269/10 | 316BK | Besenyszög, HU | 47.30 | 20.26 | 109 | Eastern | KX380094 |
| 270/10 | 317BK | Besenyszög, HU | 47.30 | 20.26 | 109 | Eastern | KX380089 |
| 271/10 | 313BK | Besenyszög, HU | 47.30 | 20.26 | 109 | Eastern | KX380021 |
| 273/10 | 355BK | Görcsöny, HU | 45.97 | 18.13 | 115 | Eastern | KX380095 |
| 589/11BK | 36BK | Gyula, HU | 46.65 | 21.28 | 108 | Eastern | KX380002 |
| 590/11BK | 37BK | Gyula, HU | 46.65 | 21.28 | 108 | Eastern | KX380073 |
| 591/11BK | 39BK | Gyula, HU | 46.65 | 21.28 | 108 | Eastern | KX380074 |
| 592/11BK | 40BK | Gyula, HU | 46.65 | 21.28 | 108 | Eastern | KX380075 |
| 1,15,19 | 02BK | Kaposvár, HU | 46.36 | 17.80 | 113 | Eastern | KX380071 |
| 1,13,19 | 03BK | Kaposvár, HU | 46.36 | 17.80 | 113 | Eastern | KX380072 |
| 1,8,12 | 01BK | Kaposvár, HU | 46.36 | 17.80 | 113 | Eastern | KX380070 |
| 307/10-E | 141BK | Keszthely, HU | 46.77 | 17.25 | 111 | Eastern | KX380006 |
| 308/10 | 143BK | Keszthely, HU | 46.77 | 17.25 | 111 | Eastern | KX380079 |
| 309/10 | 140BK | Keszthely, HU | 46.77 | 17.25 | 111 | Eastern | KX380087 |
| 300/10 | 139BK | Keszthely, HU | 46.77 | 17.25 | 111 | Eastern | KX380005 |
| 17/11BK | 243BK | Kétújfalu, HU | 45.96 | 17.71 | 117 | Eastern | KX380082 |
| 389/10 | 115BK | Kis-Balaton, HU | 46.64 | 17.14 | 112 | Eastern | KX380097 |
| 425/10 | 136BK | Kis-Balaton, HU | 46.64 | 17.14 | 112 | Eastern | KX380086 |
| 385/10 | 114BK | Kis-Balaton, HU | 46.64 | 17.14 | 112 | Eastern | KX380045 |
| E-741/11BK | 344BK | Kisvárda, HU | 48.22 | 22.08 | 103 | Eastern | KX380015 |
| E-743/11BK | 345BK | Kisvárda, HU | 48.22 | 22.08 | 103 | Eastern | KX380016 |
| E1-747/11BK | 346BK | Kisvárda, HU | 48.22 | 22.08 | 103 | Eastern | KX380088 |
| 754/11BK | 343BK | Kisvárda, HU | 48.22 | 22.08 | 103 | Eastern | KX380090 |
| 448/10 | 306BK | Lake Matty, HU | 45.80 | 18.26 | 116 | Eastern | KX380096 |
| 449/10 | 308BK | Lake Matty, HU | 45.80 | 18.26 | 116 | Eastern | KX380098 |
| 450/10 | 309BK | Lake Matty, HU | 45.80 | 18.26 | 116 | Eastern | KX380099 |
| 206/10-E2 | 164BK | Pécs, HU | 46.07 | 18.23 | 114 | Eastern | KX380008 |
| 206/10-E3 | 165BK | Pécs, HU | 46.07 | 18.23 | 114 | Eastern | KX380009 |
| 206/10-E4 | 166BK | Pécs, HU | 46.07 | 18.23 | 114 | Eastern | KX380010 |
| 207/10 | 162BK | Pécs, HU | 46.07 | 18.23 | 114 | Eastern | KX380007 |
| 785/11BK | 77BK | Sopron, HU | 47.68 | 16.58 | 89 | Eastern | KX380003 |
| 786/11BK | 76BK | Sopron, HU | 47.68 | 16.58 | 89 | Eastern | KX380076 |
| 787/11BK | 78BK | Sopron, HU | 47.68 | 16.58 | 89 | Eastern | KX380077 |
| 788/11BK | 79BK | Sopron, HU | 47.68 | 16.58 | 89 | Eastern | KX380078 |
| 789/11BK | 80BK | Sopron, HU | 47.68 | 16.58 | 89 | Eastern | KX380004 |
| 230/10-E | 177BK | Szolnok, HU | 47.16 | 20.18 | 110 | Eastern | KX380011 |
| 231/10 | 178BK | Szolnok, HU | 47.16 | 20.18 | 110 | Eastern | KX380080 |
| 231/10-E1 | 179BK | Szolnok, HU | 47.16 | 20.18 | 110 | Eastern | KX380012 |
| 231/10-E2 | 180BK | Szolnok, HU | 47.16 | 20.18 | 110 | Eastern | KX380013 |
| 679/11BK | 382BK | Tiszaszalka, HU | 48.19 | 22.31 | 104 | Eastern | KX380083 |
| 680/11BK | 383BK | Tiszaszalka, HU | 48.19 | 22.31 | 104 | Eastern | KX380084 |
| 681/11BK | 384BK | Tiszaszalka, HU | 48.19 | 22.31 | 104 | Eastern | KX380085 |
| 682/11BK | 385BK | Tiszaszalka, HU | 48.19 | 22.31 | 104 | Eastern | KX380017 |
| 683/11BK | 386BK | Tiszaszalka, HU | 48.19 | 22.31 | 104 | Eastern | KX380018 |
| E-722/11BK | 399BK | Zsarolyán, HU | 47.95 | 22.59 | 105 | Eastern | KX380019 |
| E-737/11BK | 342BK | Zsarolyán, HU | 47.95 | 22.59 | 105 | Eastern | KX380014 |
| 181/09 | 420BK | Aleksinac, SB | 43.54 | 21.72 | 123 | Eastern | KX380050 |
| 182/09 | 469BK | Aleksinac, SB | 43.54 | 21.72 | 123 | Eastern | KX380032 |
| 183/09 | 422BK | Aleksinac, SB | 43.54 | 21.72 | 123 | Eastern | KX380023 |
| 184/09 | 423BK | Aleksinac, SB | 43.54 | 21.72 | 123 | Eastern | KX380024 |
| 186/10 | 462BK | Dimitrovgrad, SB | 43.01 | 22.77 | 139 | Balkan | KX380029 |
| 185/10 | 463BK | Dimitrovgrad, SB | 43.01 | 22.77 | 139 | Balkan | KX380053 |
| 188/10 | 464BK | Dimitrovgrad, SB | 43.01 | 22.77 | 139 | Balkan | KX380030 |
| 187/10 | 465BK | Dimitrovgrad, SB | 43.01 | 22.77 | 139 | Balkan | KX380031 |
| 223/09 | 495BK | Knjaževac, SB | 43.57 | 22.25 | 138 | Balkan | KX380033 |
| 224/09 | 496BK | Knjaževac, SB | 43.57 | 22.25 | 138 | Balkan | KX380049 |
| 225/09 | 497BK | Knjaževac, SB | 43.57 | 22.25 | 138 | Balkan | KX380034 |
| 226/09 | 498BK | Knjaževac, SB | 43.57 | 22.25 | 138 | Eastern | KX380035 |
| 65/09 | 430BK | Vračev Gaj, SB | 44.88 | 21.32 | 122 | Eastern | KX380048 |
| E1-65/09 | 431BK | Vračev Gaj, SB | 44.88 | 21.32 | 122 | Eastern | KX380025 |
| E2-65/09 | 432BK | Vračev Gaj, SB | 44.88 | 21.32 | 122 | Eastern | KX380026 |
| E3-65/09 | 433BK | Vračev Gaj, SB | 44.88 | 21.32 | 122 | Eastern | KX380027 |
| E4-65/09 | 434BK | Vračev Gaj, SB | 47.95 | 22.59 | 105 | Eastern | KX380028 |
