# Supplementary material for: Between the Balkans and the Baltic: Phylogeography of a Common Vole Mitochondrial DNA Lineage Limited to Central Europe
Source: PLoS One. 2016 Dec 16;11(12):e0168621. doi: 10.1371/journal.pone.0168621 (PMC5161492; doi:10.1371/journal.pone.0168621)
Supplement: S2 Table — The full list of previously published cytb sequences that were used in this study are available in S1, S2 and S4 Tables in [30]. The numbers in square brackets are consistent with the References section. The reference not used in the References section is marked by ‘*’ and its full description is given below the table. (DOCX) [file pone.0168621.s008.docx]

**S2 Table. List of those collection localities from central and eastern Europe mapped in Fig 1B which provided previously published data for this study but which were not used to generate new cyt*b* or microsatellite data.** The full list of previously published cyt*b* sequences that were used in this study are available in S1, S2 and S4 Tables in [30]. The numbers in square brackets are consistent with the References section. The reference not used in Reference section is marked by ‘*’ and its full description is given below the table.

| **Map reference**  **(Fig 1B)** | **Locality** | **References** |
| --- | --- | --- |
| 9 | Chernobyl | Barker et al. 1996* |
| 1  4  32  90  91  98 | Nuijamaa  Vladimir  WilczaGóra  Nagycsány  VelkéKosihy  Stebník | Haynes et al. 2003 [24] |
| 121  124  125  126  127  128  129  130  131  132  133  134  136  137 | Slano Kopovo  Mt. Suva planina  Mt. Komovi  Mt. Bjelašnica  Morine  Mt. Zelengora  Gacko  Kupres  Blidinje  Tomislavgrad  Mt. Šator  Bosanski Petrovac  Kranj  Ljubljana | Bužan et al. 2010 [29] |
| 79, 84, 86-88 | Czech Republic | Tougard et al. 2013 [72] |
| 10  25  37  41  47  50  51  52  53  140 | Cherkassy  Miłomłyn  Popówka  Włocławek  Łowicz  Warka  Grójec  Kozienice  Januszno  Buchak | Stojak et al. 2015 [30] |
| 22  29  58 | Kadyny  Mikaszówka  Rzepin | Stojak et al. 2016 [34] |

* Barker RJ, van den Bussche RA, Wright AJ, Wiggins LE, Hamilton MJ, Reat EP et al. High levels of genetic change in rodents of Chernobyl. Nature. 1996; 380: 707–708.
