# Supplementary material for: Between the Balkans and the Baltic: Phylogeography of a Common Vole Mitochondrial DNA Lineage Limited to Central Europe
Source: PLoS One. 2016 Dec 16;11(12):e0168621. doi: 10.1371/journal.pone.0168621 (PMC5161492; doi:10.1371/journal.pone.0168621)
Supplement: S3 Table — Samples used in the previous study of Stojak et al. [34] are marked by ‘#’. Significant values for FIS are given after Bonferroni correction and marked by ‘*’. Only samples with ≥ 5 individuals were used for calculations. (DOCX) [file pone.0168621.s009.docx]

**S3 Table. Diversity indices for samples of *Microtus arvalis* used in this study for microsatellite analysis including sampling locality with geographic coordinates, sample size (n), observed (H_O_) and expected (H_E_) heterozygosity, allelic richness (AR) and the inbreeding coefficient (F_IS_).** Samples used in the previous study of Stojak et al. [34] are marked by ‘#’. Significant values for F_IS_ are given after Bonferroni correction and marked by ‘*’. Only samples with ≥ 5 individuals were used for calculations.

| **Sampling locality** | **Map reference (see Fig 1B)** | **Symbol** | **n** | **Longitude (E)** | **Latitude (N)** | **H_O_** | **H_E_** | **AR** | **F_IS_** |
| --- | --- | --- | --- | --- | --- | --- | --- | --- | --- |
| Aleksinac, SB | 123 | ALE | 13 | 21.72 | 43.54 | 0.817 | 0.861 | 5.86 | 0.052 |
| Besenyszög, HU | 109 | BES | 14 | 20.26 | 47.30 | 0.795 | 0.845 | 5.91 | 0.062 |
| Białowieża, PL | 36 | BIA | 8 | 23.87 | 52.70 | 0.810 | 0.853 | 6.24 | 0.053 |
| Bližna, CZ | 82 | BLI | 14 | 14.10 | 48.72 | 0.786 | 0.877 | 6.54 | 0.107 |
| Česky Dub, CZ | 77 | CZD | 13 | 15.00 | 50.66 | 0.788 | 0.800 | 5.39 | 0.015 |
| Czernogolowka, RU | 3 | CER | 10 | 38.39 | 56.01 | 0.863 | 0.830 | 5.66 | -0.042 |
| Kaposvár, HU | 113 | KAP | 14 | 17.80 | 46.36 | 0.723 | 0.857 | 6.47 | 0.162* |
| Konin, PL | 42 | KON | 8 | 18.25 | 52.22 | 0.766 | 0.826 | 6.23 | 0.078 |
| Koprivnice, CZ | 92 | KOP | 14 | 18.14 | 49.60 | 0.893 | 0.823 | 5.58 | -0.089 |
| Kubinskoye, RU | 2 | KUB | 14 | 36.40 | 55.28 | 0.770 | 0.833 | 5.80 | 0.079 |
| Ljubljansko Barje, SLO | 118 | LJU | 14 | 14.40 | 45.99 | 0.759 | 0.798 | 5.30 | 0.050 |
| Novy Drahov, CZ | 81 | NDR | 15 | 12.39 | 50.14 | 0.774 | 0.861 | 6.44 | 0.104 |
| Pécs, HU | 114 | PCS | 13 | 18.23 | 46.07 | 0.769 | 0.857 | 6.33 | 0.107 |
| Reporyje, CZ | 78 | REP | 14 | 14.17 | 50.01 | 0.777 | 0.830 | 6.26 | 0.066 |
| Sopron, HU | 89 | SOP | 12 | 16.58 | 47.68 | 0.791 | 0.886 | 7.01 | 0.112 |
| Tiszaszalka, HU | 104 | TSZ | 14 | 22.31 | 48.19 | 0.821 | 0.915 | 7.22 | 0.105 |
| Urwitałt, PL | 26 | URW | 15 | 21.64 | 53.81 | 0.792 | 0.843 | 6.20 | 0.063 |
| Vračev Gaj, SB | 122 | VRG | 14 | 21.32 | 44.88 | 0.798 | 0.867 | 6.46 | 0.082 |
| Wojtkówka, PL | 100 | WOJ | 15 | 22.56 | 49.56 | 0.739 | 0.893 | 6.84 | 0.178* |
| Januszno, PL | 54 | JAN | 15 | 21.51 | 51.49 | 0.754 | 0.857 | 6.33 | 0.124* |
| Zgierz, PL | 45 | LOD | 7 | 18.96 | 51.89 | 0.893 | 0.768 | 4.49 | -0.179 |
| Zielątkowo, PL | 68 | ZEL | 6 | 16.80 | 52.55 | 0.708 | 0.799 | 5.71 | 0.124 |
| Skwierzyna, PL | 63 | SKW | 12 | 15.51 | 52.60 | 0.667 | 0.855 | 6.29 | 0.228* |
| Świdnica, PL | 75 | SWI | 7 | 16.66 | 50.91 | 0.804 | 0.889 | 6.59 | 0.103 |
| Sochaczew, PL | 49 | NDM | 16 | 20.24 | 52.23 | 0.821 | 0.889 | 6.62 | 0.079 |
| KrosnoOdrzańskie, PL# | 60 | KRO | 15 | 14.97 | 52.24 | 0.786 | 0.854 | 6.28 | 0.083 |
| Pułkownikówka, PL# | 20 | PLK | 12 | 19.28 | 54.21 | 0.833 | 0.853 | 6.24 | 0.024 |
| Pomorze, PL# | 28 | POM | 11 | 23.38 | 54.05 | 0.773 | 0.836 | 6.04 | 0.079 |
| Bobolice, PL# | 15 | BOB | 6 | 16.59 | 53.95 | 0.667 | 0.839 | 5.62 | 0.221 |
| Bolewice, PL# | 69 | BLW | 3 | 16.12 | 52.40 | - | - | - | - |
| Cisna, PL# | 101 | CIS | 10 | 22.33 | 49.21 | 0.832 | 0.888 | 7.02 | 0.066 |
| Darżlubie, PL# | 19 | DRZ | 6 | 18.33 | 54.70 | 0.708 | 0.816 | 5.61 | 0.144 |
| GórowoIławieckie, PL# | 23 | GIL | 7 | 20.49 | 54.29 | 0.732 | 0.864 | 6.43 | 0.163 |
| Goszcz, PL# | 59 | GSZ | 3 | 17.48 | 51.40 | - | - | - | - |
| Iława, PL# | 24 | ILA | 5 | 19.57 | 53.60 | 0.925 | 0.797 | 4.88 | -0.184 |
| Kryńszczak, PL# | 39 | KRY | 9 | 22.36 | 51.99 | 0.778 | 0.865 | 6.44 | 0.107 |
| Nurzec, PL# | 34 | NUR | 3 | 22.43 | 52.62 | - | - | - | - |
| RudaRóżaniecka, PL# | 57 | RRO | 5 | 23.18 | 50.32 | 0.825 | 0.842 | 6.88 | 0.022 |
| Sobibór, PL# | 38 | SOB | 10 | 23.64 | 51.48 | 0.800 | 0.916 | 7.47 | 0.133* |
| Świętokrzyski NP, PL# | 56 | SPN | 8 | 20.91 | 50.90 | 0.750 | 0.828 | 5.87 | 0.100 |
| Świerzawa, PL# | 76 | SRZ | 9 | 15.89 | 51.01 | 0.750 | 0.825 | 5.84 | 0.096 |
| Trzebieszki, PL# | 18 | TRB | 11 | 16.62 | 53.36 | 0.761 | 0.865 | 6.17 | 0.125 |
| Wierzchlas, PL# | 44 | WCH | 5 | 18.11 | 53.52 | 0.750 | 0.881 | 6.50 | 0.164 |
| Wiśniowa, PL# | 96 | WSN | 6 | 20.09 | 49.78 | 0.833 | 0.797 | 5.85 | -0.050 |
| Wojsław, PL# | 93 | WSL | 5 | 21.46 | 50.26 | 0.850 | 0.881 | 6.13 | 0.039 |
| Wschowa, PL# | 62 | WSH | 5 | 16.32 | 51.81 | 0.800 | 0.892 | 6.63 | 0.114 |
| Zagożdżon, PL# | 55 | ZAG | 3 | 21.44 | 51.53 | - | - | - | - |
| Złocieniec, PL# | 16 | ZLO | 3 | 16.01 | 53.53 | - | - | - | - |
| Żytkiejmy, PL# | 27 | ZTK | 4 | 22.70 | 54.35 | - | - | - | - |
| Bogdaniec, PL# | 66 | BGD | 3 | 15.07 | 52.69 | - | - | - | - |
| Międzychód, PL# | 67 | MDH | 9 | 15.89 | 52.60 | 0.847 | 0.842 | 5.97 | -0.006 |
| Zielona, PL# | 94 | ZLN | 3 | 17.95 | 50.65 | - | - | - | - |
| CzarnaBiałostocka, PL# | 31 | CZB | 1 | 23.29 | 53.30 | - | - | - | - |
| Krzystkowice, PL# | 61 | KRZ | 1 | 15.24 | 51.80 | - | - | - | - |
| Łochów, PL# | 33 | LCH | 1 | 21.68 | 52.53 | - | - | - | - |
| Przyborów, PL# | 43 | PBR | 2 | 16.46 | 51.44 | - | - | - | - |
| Rajgród, PL# | 30 | RAJ | 1 | 22.70 | 53.73 | - | - | - | - |
| Rogalice, PL# | 74 | RGL | 1 | 17.61 | 50.96 | - | - | - | - |
| Ryjewo, PL# | 21 | RYJ | 1 | 18.96 | 53.84 | - | - | - | - |
| StaryKraków, PL# | 14 | SKR | 2 | 16.62 | 54.44 | - | - | - | - |
| Szprotawa, PL# | 70 | SPR | 1 | 15.54 | 51.57 | - | - | - | - |

SB-Serbia. HU-Hungary. PL-Poland. CZ-Czech Republic. RU-Russia. SLO-Slovenia
