# Supplementary material for: Between the Balkans and the Baltic: Phylogeography of a Common Vole Mitochondrial DNA Lineage Limited to Central Europe
Source: PLoS One. 2016 Dec 16;11(12):e0168621. doi: 10.1371/journal.pone.0168621 (PMC5161492; doi:10.1371/journal.pone.0168621)
Supplement: S4 Table — (DOCX) [file pone.0168621.s010.docx]

**S4 Table. Distribution of nucleotide polymorphisms with data organized by individual nucleotides (upper panel) and triplets (lower panel) in the *Microtus arvalis* cytochrome *b* sequences from newly-collected samples from central Europe.**

| Codon position | Proportion of  polymorphic sites | Number of transitions | Number of transversions |
| --- | --- | --- | --- |
| First  Second  Third  Overall | 0.060 (23/380)  0.008 (3/380)  0.245 (93/380)  0.104 (119/1140) | 0.053 (20/380)  0.005 (2/380)  0.221 (84/380)  0.093 (106/1140) | 0.011 (4/380)  0.003 (1/380)  0.21 (8/380)  0.011 (13/1140) |
| Protein domains | Proportion of  polymorphic sites | Number of synonymous mutations | Number of non-synonymous mutations |
| Transmembrane  Intermembrane  Matrix  Overall | 0.276 (52/188)  0.342 (39/114)  0.359 (28/78)  0.313 (119/380) | 0.218 (41/188)  0.307 (35/114)  0.295 (23/78)  0.261 (99/380) | 0.059 (11/188)  0.035 (4/114)  0.064 (5/78)  0.053 (20/380) |
