# Supplementary material for: Between the Balkans and the Baltic: Phylogeography of a Common Vole Mitochondrial DNA Lineage Limited to Central Europe
Source: PLoS One. 2016 Dec 16;11(12):e0168621. doi: 10.1371/journal.pone.0168621 (PMC5161492; doi:10.1371/journal.pone.0168621)
Supplement: S6 Table — (DOCX) [file pone.0168621.s012.docx]

**S6 Table. Characteristics of loci used in the microsatellite analysis of the whole *Microtus arvalis* dataset including observed (H_O_) and expected (H_E_) heterozygosity and null alleles.**

| **Locus** | **H_O_** | **H_E_** | **Null Alleles** |
| --- | --- | --- | --- |
| Ma25 | 0.903 | 0.925 | No |
| Ma36 | 0.880 | 0.920 | No |
| Ma68 | 0.874 | 0.891 | No |
| MSM6 | 0.823 | 0.839 | No |
| Ma75 | 0.671 | 0.881 | No |
| Mag6 | 0.535 | 0.581 | No |
| Ma29 | 0.861 | 0.887 | No |
| Moe6 | 0.759 | 0.874 | Yes |
