# Supplementary material for: Between the Balkans and the Baltic: Phylogeography of a Common Vole Mitochondrial DNA Lineage Limited to Central Europe
Source: PLoS One. 2016 Dec 16;11(12):e0168621. doi: 10.1371/journal.pone.0168621 (PMC5161492; doi:10.1371/journal.pone.0168621)
Supplement: S7 Table — Population labels are given according to S3 Table. Significant values are given after Bonferroni correction and marked by ‘*’. (DOCX) [file pone.0168621.s013.docx]

**S7 Table (in three parts). Estimates of multilocus F_ST_ between all population pairs of *Microtus arvalis* based on microsatellites.** Population labels are given according to Table S3. Significant values are given after Bonferroni correction and marked by ‘*’.

|  | BES BIA BLI BOB CER CIS CZD DRZ GIL ILA JAN KAP KON KOP KRO KRY KUB LJU LOD |
| --- | --- |
| ALE  BES  BIA  BLI  BOB  CER  CIS  CZD  DRZ  GIL  ILA  JAN  KAP  KON  KOP  KRO  KRY  KUB  LJU | 0.0710* 0.0566 0.0499* 0.0662 0.0682* 0.0503 0.0620* 0.0707 0.0581* 0.0850 0.0619* 0.0607* 0.0762* 0.0821* 0.0508* 0.0446 0.0577* 0.0773* 0.1043  0.0606* 0.0611* 0.0543 0.0591* 0.0470* 0.0734* 0.0728* 0.0287 0.0751 0.0574* 0.0539* 0.0294 0.0690* 0.0456* 0.0318 0.0548* 0.0569* 0.0679  0.0449 0.0541 0.0693 0.0238 0.0765 0.0329 0.0355 0.0812 0.0534* 0.0314 0.0446 0.0678 0.0382 0.0145 0.0438 0.0552 0.0937  0.0098 0.0654* 0.0344 0.0501 0.0274 0.0225 0.0654 0.0351* 0.0139 0.0385 0.0511* 0.0278 0.0213 0.0580* 0.0719* 0.0656  0.0744 0.0441 0.0608 0.0146 -0.0010 0.0870 0.0512 0.0194 0.0481 0.0722 0.0343 0.0306 0.0565 0.0907 0.1019  0.0527 0.0513 0.0919 0.0650 0.0559 0.0501 0.0659* 0.0686 0.1091* 0.0612 0.0548* 0.0670* 0.0872* 0.1199  0.0587 0.0253 0.0247 0.0657 0.0212 0.0251 0.0271 0.0474 0.0255 0.0105 0.0523* 0.0581 0.0883  0.0821 0.0652* 0.0812 0.0740* 0.0680 0.0695 0.0999* 0.0565 0.0486 0.0752* 0.0922* 0.0783  -0.0092 0.0765 0.0278 0.0197 0.0450 0.0738 0.0274 0.0158 0.0372 0.0828 0.1204  0.0570 0.0256 -0.0002 0.0237 0.0459 0.0131 0.0142 0.0272 0.0400 0.0766  0.0126 0.0629 0.0678 0.0879 0.0586 0.0577 0.0848 0.0628 0.1204  0.0255 0.0415 0.0716* 0.0441* 0.0272 0.0631* 0.0640* 0.0967  0.0247 0.0645* 0.0244 0.0193 0.0565* 0.0635* 0.0824  0.0471 0.0187 -0.0062 0.0369 0.0364 0.0799  0.0463 0.0424 0.0644* 0.0697* 0.0853  -0.0009 0.0392* 0.0449* 0.0759  0.0197 0.0450 0.0711  0.0694* 0.1064*  0.0782* |

Continuation of S7 Table, part 2

|  | MDH NDM NDR PCS PLK POM REP RRO SKW SOB SOP SPN SRZ SWI TRB TSZ URW VRG WCH |
| --- | --- |
| ALE  BES  BIA  BLI  BOB  CER  CIS  CZD  DRZ  GIL  ILA  JAN  KAP  KON  KOP  KRO  KRY  KUB  LJU  LOD  MDH  NDM  NDR  PCS  PLK  POM  REP  RRO  SKW  SOB  SOP  SPN  SRZ  SWI  TRB  TSZ  URW  VRG | 0.0609* 0.0387* 0.0504* 0.0617* 0.0541* 0.0639* 0.0619* 0.0557 0.0620* 0.0449 0.0408* 0.0565 0.0591 0.0363 0.0405* 0.0486* 0.0492* 0.0552* 0.0390  0.0364 0.0446* 0.0404* 0.0318* 0.0414 0.0372 0.0543* 0.0266 0.0114 0.0262 0.0395 0.0439 0.0411 0.0318 0.0538* 0.0318 0.0434* 0.0260 0.0279  0.0505 0.0316 0.0228 0.0487 0.0301 0.0220 0.0472 0.0195 0.0200 0.0342 0.0276 0.0594 0.0387 -0.0051 0.0139 0.0143 0.0358 0.0433 0.0332  0.0468* 0.0368* 0.0375* 0.0327 0.0193 0.0468* 0.0246 0.0199 0.0394 0.0256 0.0177 0.0479 0.0409* 0.0181 0.0468* 0.0217 0.0414 0.0124 0.0270  0.0671 0.0439* 0.0410 0.0405 0.0391 0.0499 0.0344 0.0149 0.0470 0.0369 0.0184 0.0605 0.0325 0.0225 0.0539 0.0321 0.0621 0.0209 0.0283  0.0800 0.0558* 0.0632* 0.0459 0.0610* 0.0800* 0.0738 0.0742 0.0562* 0.0420 0.0398 0.0661* 0.0528 0.0527 0.0619* 0.0476* 0.0543* 0.0608* 0.0584  0.0384 0.0362* 0.0275 0.0464* 0.0220 0.0307 0.0358* 0.0054 0.0239 0.0172 0.0146 0.0654 0.0526 0.0086 0.0258 0.0184 0.0277 0.0242 0.0192  0.0636 0.0704* 0.0499 0.0592 0.0618* 0.0719 0.0613 0.0637 0.0625 0.0604 0.0499 0.0695 0.0618 0.0508 0.0660* 0.0665* 0.0562* 0.0366 0.0525  0.0561 0.0387 0.0277 0.0564 0.0316 0.0471 0.0239 0.0294 0.0384 0.0354 0.0134 0.0873 0.0667 0.0160 0.0508 0.0383 0.0525 0.0236 0.0400  0.0316 0.0252 0.0119 0.0307 0.0235 0.0304 0.0252 -0.0075 0.0141 0.0197 0.0061 0.0585 0.0346 0.0126 0.0292 0.0191 0.0407 0.0126 0.0277  0.0759 0.0587 0.0560 0.0588 0.0703 0.0706 0.0732 0.0780 0.0660 0.0499 0.0397 0.0858 0.0686 0.0467 0.0771 0.0577 0.0479 0.0476 0.0664  0.0504 0.0308* 0.0416* 0.0422 0.0473* 0.0586* 0.0374 0.0350 0.0448* 0.0320 0.0149 0.0791* 0.0456 0.0259 0.0411* 0.0288 0.0376* 0.0301* 0.0381  0.0498* 0.0427* 0.0299* 0.0470 0.0252 0.0416* 0.0304 0.0112 0.0370* 0.0249 0.0100 0.0754* 0.0447* 0.0160 0.0509* 0.0212 0.0396* 0.0212 0.0426  0.0213 0.0388 0.0170 0.0133 0.0148 0.0305 -0.0058 0.0172 0.0277 0.0158 0.0130 0.0575 0.0236 0.0065 0.0376* 0.0285 0.0195 0.0186 0.0340  0.0631* 0.0604* 0.0593* 0.0603* 0.0589* 0.0709* 0.0675* 0.0549 0.0626* 0.0589* 0.0561* 0.0746 0.0777* 0.0454 0.0701* 0.0490* 0.0647* 0.0539* 0.0698  0.0422* 0.0446* 0.0314* 0.0359 0.0213 0.0253 0.0194 0.0187 0.0310 0.0293 0.0257 0.0428 0.0378 0.0114 0.0425* 0.0231 0.0301 0.0224 0.0390  0.0286 0.0371* 0.0193 0.0226 0.0029 0.0254* 0.0027 0.0073 0.0163 0.0105 0.0043 0.0335 0.0201 -0.0103 0.0085 0.0169 0.0090 0.0102 0.0095  0.0485* 0.0442* 0.0411 0.0539 0.0402* 0.0451* 0.0369* 0.0364 0.0496* 0.0488* 0.0254 0.0751* 0.0553* 0.0352 0.0476* 0.0437* 0.0565* 0.0494* 0.0475  0.0415 0.0588* 0.0422* 0.0447* 0.0538* 0.0419* 0.0497* 0.0390 0.0301* 0.0327 0.0452* 0.0500 0.0259* 0.0466 0.0559* 0.0471* 0.0561* 0.0468* 0.0726  0.0385 0.0948* 0.0821* 0.0805* 0.0692 0.0688 0.0810* 0.0964 0.0709 0.0716 0.0751 0.0976 0.0867 0.0827 0.0921 0.0764* 0.0960* 0.0447 0.0991  0.0317 0.0334 0.0423 0.0294 0.0252 0.0293 0.0343 0.0368* 0.0340 0.0213 0.0788 0.0387* 0.0207 0.0396 0.0313 0.0542* 0.0210 0.0434  0.0110 0.0306 0.0293 0.0337 0.0428* 0.0374 0.0356 0.0299 0.0233 0.0543* 0.0440 0.0036 0.0309* 0.0137 0.0319 0.0319* 0.0183  0.0196 0.0104 0.0257 0.0325 0.0192 0.0270 0.0180 0.0192 0.0460 0.0313 -0.0033 0.0244 0.0200 0.0253 0.0105 0.0095  0.0110 0.0414 0.0258 0.0377 0.0281 0.0107 0.0303 0.0307 0.0189 0.0230 0.0415* 0.0303 0.0243 0.0178 0.0321  0.0097 0.0215 0.0232 0.0309 0.0147 0.0216 0.0359 0.0324* 0.0062 0.0347* 0.0178 0.0197 -0.0010 0.0273  0.0270 0.0046 0.0255 0.0349 0.0296* 0.0426 0.0386* 0.0118 0.0398* 0.0255 0.0387* 0.0266 0.0373  0.0228 0.0406* 0.0323 0.0180 0.0684* 0.0355 0.0191 0.0350* 0.0348* 0.0335 0.0211 0.0510  -0.0134 0.0169 0.0017 0.0394 0.0069 0.0047 0.0207 0.0122 0.0224 0.0210 0.0152  0.0016 0.0240 0.0293 0.0101 0.0118 0.0264 0.0162 0.0303 0.0193 0.0135  0.0091 0.0230 0.0185 0.0095 0.0305 0.0076 0.0240 0.0103 0.0208  0.0559 0.0208 0.0103 0.0300* 0.0075 0.0249 0.0141 0.0176  0.0253 0.0295 0.0611* 0.0511 0.0364 0.0389 0.0333  0.0197 0.0341* 0.0332 0.0336 0.0298 0.0334  -0.0014 0.0035 0.0088 0.0099 0.0040  0.0182 0.0364* 0.0340* 0.0163  0.0275 0.0148 0.0122  0.0325* 0.0335  0.0149 |

Continuation of S7 Table, part 3 (end of Table)

|  | WOJ WSH WSL WSN ZEL |
| --- | --- |
| ALE  BES  BIA  BLI  BOB  CER  CIS  CZD  DRZ  GIL  ILA  JAN  KAP  KON  KOP  KRO  KRY  KUB  LJU  LOD  MDH  NDM  NDR  PCS  PLK  POM  REP  RRO  SKW  SOB  SOP  SPN  SRZ  SWI  TRB  TSZ  URW  VRG  WCH  WOJ  WSH  WSL  WSN | 0.0363* 0.0312 0.0608 0.0820 0.0841*  0.0406* 0.0327 0.0355 0.0590 0.0571*  0.0281 0.0216 0.0348 0.0664 0.0611  0.0280* 0.0145 0.0410 0.0398 0.0522*  0.0303 0.0165 0.0601 0.0429 0.0422  0.0429* 0.0419 0.0510 0.0964 0.0935  0.0105 0.0132 0.0240 0.0405 0.0529  0.0309 0.0518 0.0799 0.0897 0.0781  0.0209 0.0025 0.0621 0.0204 0.0398  0.0159 -0.0205 0.0381 0.0270 0.0172  0.0497 0.0334 0.0861 0.0923 0.0811  0.0287* 0.0146 0.0523 0.0505 0.0536  0.0289 0.0125 0.0389 0.0265 0.0397*  0.0304 0.0174 0.0415 0.0266 0.0283  0.0682* 0.0451 0.0789 0.0925* 0.0815  0.0248 0.0066 0.0360 0.0233 0.0344  0.0065 -0.0013 0.0216 0.0159 0.0381  0.0455* 0.0390 0.0714 0.0583 0.0667  0.0437* 0.0177 0.0263 0.0795 0.0424  0.0843 0.0635 0.0649 0.1288 0.0662  0.0338* 0.0270 0.0441 0.0617 0.0472  0.0304* 0.0133 0.0417 0.0557 0.0515  0.0170 -0.0088 0.0405 0.0313 0.0205  0.0282 0.0098 0.0288 0.0511 0.0313  0.0225 0.0080 0.0167 0.0298 0.0436  0.0402* 0.0124 0.0189 0.0471* 0.0413  0.0300 0.0130 0.0542 0.0149 0.0273  0.0127 0.0172 0.0278 0.0474 0.0499  0.0164 0.0145 -0.0064 0.0348 0.0265  0.0054 -0.0031 -0.0030 0.0366 0.0305  0.0132 -0.0032 0.0288 0.0197 0.0295  0.0362 0.0281 0.0202 0.0973 0.0779  0.0169 0.0109 0.0248 0.0588 0.0324  0.0100 -0.0081 0.0356 0.0194 0.0385  0.0218 0.0166 0.0418 0.0618 0.0517*  0.0144 -0.0037 0.0162 0.0417 0.0408  0.0207 0.0193 0.0490 0.0391 0.0540  0.0187 -0.0052 0.0237 0.0171 0.0115  -0.0044 -0.0098 0.0475 0.0440 0.0510  0.0048 0.0216 0.0289 0.0344  0.0192 0.0107 0.0029  0.0707 0.0410  0.0286 |
